# Epigenetic Basis of Regeneration: Analysis of Genomic DNA Methylation Profiles in the MRL/MpJ Mouse

**DOI:** 10.1093/dnares/dst034

**Published:** 2013-08-08

**Authors:** Bartosz Górnikiewicz, Anna Ronowicz, Justyna Podolak, Piotr Madanecki, Anna Stanisławska-Sachadyn, PaweŁ Sachadyn

**Affiliations:** 1 Department of Microbiology, Gdańsk University of Technology, Gdańsk, Poland; 2 Department of Biology and Pharmaceutical Botany, Medical University of Gdańsk, Gdańsk, Poland; 3 Department of Biology and Genetics, Medical University of Gdańsk, Gdańsk, Poland

**Keywords:** MRL/MpJ mouse, regeneration, DNA methylation, epigenetics, genome-wide DNA methylation profiling

## Abstract

Epigenetic regulation plays essential role in cell differentiation and dedifferentiation, which are the intrinsic processes involved in regeneration. To investigate the epigenetic basis of regeneration capacity, we choose DNA methylation as one of the most important epigenetic mechanisms and the MRL/MpJ mouse as a model of mammalian regeneration known to exhibit enhanced regeneration response in different organs. We report the comparative analysis of genomic DNA methylation profiles of the MRL/MpJ and the control C57BL/6J mouse. Methylated DNA immunoprecipitation followed by microarray analysis using the Nimblegen ‘3 × 720 K CpG Island Plus RefSeq Promoter’ platform was applied in order to carry out genome-wide DNA methylation profiling covering 20 404 promoter regions. We identified hundreds of hypo- and hypermethylated genes and CpG islands in the heart, liver, and spleen, and 37 of them in the three tissues. Decreased inter-tissue diversification and the shift of DNA methylation balance upstream the genes distinguish the genomic methylation patterns of the MRL/MpJ mouse from the C57BL/6J. Homeobox genes and a number of other genes involved in embryonic morphogenesis are significantly overrepresented among the genes hypomethylated in the MRL/MpJ mouse. These findings indicate that epigenetic patterning might be a likely molecular basis of regeneration capability in the MRL/MpJ mouse.

## 1. Introduction

The phenomenon of regeneration has become the object of extensive research in recent years. The investigations on the molecular basis of regeneration in different models were rarely focused on epigenetic aspects, while it seems that these are the epigenetic mechanisms which could play a key role in both maintaining the capacity for regeneration and remodelling involved in the regenerative response. All organs in an organism originate from a single cell (zygote), which clearly indicates that a single genome contains the information needed to grow organs and tissues, and possibly to restore lost and injured structures. This information is stored through the whole life. However, the expression of this information is gradually repressed during development, with epigenetic mechanisms including DNA methylation playing an essential part.

Although the direct evidence on the involvement of DNA methylation in regeneration is scarce, a few remarkable findings are worth mentioning including the role of the *Shh* gene methylation in amphibian limb regeneration^1^ and the changes of DNA methylation status in the *Gadd45a* promoter found during folate-induced regeneration response in the rodent spinal cord.^2^ To our knowledge, no analysis of genome-wide DNA methylation profiles related to regeneration has been reported so far.

The MRL/MpJ mouse, an inbred laboratory strain of *Mus musculus*, emerged as the model of mammalian regeneration in the 1990s when the unusual phenomenon of regenerative ear-hole closure was discovered^3^ to be followed by the observations of enhanced regenerative response in other tissues including cornea,^4^ retina,^5,6^ digit tips,^7,8^ heart,^9^ articular cartilage,^10^ and spinal cord. The ability to close 2 mm through-and-through ear holes in the ear pinnae, which are used for a lifelong animal marking, clearly distinguishes the MRL/MpJ from most other mouse strains, although it is shared with the LG/J mouse, the MRL/MpJ's major ancestral strain.^11^ The response to injury in the MRL/MpJ mouse does not result in scar formation, but in complete restoration of excised fragment with normal tissue architecture, including blood vessels, hair follicles, sebaceous glands, and cartilage, that occurs within 30 days.^3^ Since this process requires reinstatement of different types of tissues, it resembles epimorphic regeneration observed in amphibians. One important feature of injury response in the MRL/MpJ mouse is increased activity of matrix metalloproteinases that decompose the extracellular matrix breaking the basal lamina, a layer that initially covers the wound surface. This process allows the formation of the blastema-like structure of undifferentiated cells that drive the process of regeneration.^12^ Another important feature of the MRL/MpJ mouse is its abnormal cell cycle profile with accumulation of the cells in the G2/M phase. This hallmark has been connected with the deficiency of p21, an inhibitor of cell cycle progression, that is not detected in the tissues of the MRL/MpJ mouse even after γ-irradiation. Although the regeneration abilities of the MRL/MpJ expand themselves on other tissues, they do not reach as spectacular effects as those observed in urodele amphibians which restore lost limbs^13^ and regenerate as serious injuries as the complete transection of the spinal cord^14^ and partial heart resection.^15^ In the MRL/MpJ mouse, lost digits do not re-grow,^8^ heart regeneration is rather limited,^9,16,17^ and the heart does not heal in some types of injuries.^18–22^ The regenerative phenotype of the MRL/MpJ mouse has been found to be multigenic.^23^ It is also remarkable that increased regenerative response in the MRL/MpJ mouse is found in different tissues, and the strain maintains selected features of embryonic metabolism in adults including the enhanced expression of key stem cell markers, *Sox2* and *Nanog*.^24,25^

The object of the presented study is the comparative analysis of the genome-wide DNA methylation profiles in the MRL/MpJ “healer” mouse, using the C57BL/6J mouse as the reference. In our attempt to understand the epigenetic basis of regeneration, we would like to identify the differentially methylated genes and to evaluate their possible association with the regenerative capacity of the MRL/MpJ mouse. The study was designed to analyse global DNA methylation profiles in heart tissue, as it is known to have poor, if any, regenerative capacity, in order to refer to the liver, which has a strong regenerative potential, in addition to spleen, selected for its involvement in cell-mediated pathways of the immune system. As the regenerating tissues are difficult to obtain in sufficient amounts and the DNA methylation patterns in cultured cell lines are prone to change, we would like to focus the study on the normal tissues, thus examining the basis of regeneration rather than the regenerative response.

## 2. Materials and methods

### 2.1. Tissue samples and nucleic acid extraction

Tissue samples (hearts, livers, and spleens) from 5-week-old males and 8-week-old females of MRL/MpJ (stock #000486) and C57BL/6J (stock #000664) murine strains were purchased from the Jackson Laboratory. The samples from 8 week-old females were not used for the microarray analyses, but for the validation experiments only. Genomic DNA was isolated using the DNeasy Blood &Tissue Kit (QIAGEN, cat. no. 69504) and RNA with the RNeasy Mini Kit (QIAGEN, cat. no. 74104) according to the manufacturer's protocols. Tissues were delivered in RNAlater stablilization reagent (QIAGEN, cat. no. 76104) and were disrupted in liquid nitrogen before nucleic acids isolation.

### 2.2. Methylated DNA immunoprecipitation

Methylated DNA immunoprecipitation (MeDIP) was performed according to the modified Weber's protocol.^26^ Equal amounts of genomic DNA from three individuals were pooled for each strain and tissue. Each pooled sample (7 µg) was diluted in 150 µl of water and randomly sheared by sonication to generate fragments between 300 and 1000 bp. A 4-µg portion of sonicated DNA was diluted in 450 µL of 10 mM Tris–HCl, pH 8.0, heat denatured at 98°C for 10 min and immediately cooled on ice for 10 min. A portion of the untreated, sonicated DNA was left to serve as the input control. The CpG-methylated DNA fragments were precipitated with anti-5-methyl cytidine antibody (Eurogentec cat. no. BI-MECY-1000), and the antibody–DNA complexes were captured with Dynabeads (Invitrogen, cat. no. 11202). The collected beads were washed in order to remove non-specifically-bound DNA, following the treatment with proteinase K for 20–24 h at 50°C in order to remove proteins. The CpG-methylated DNA was extracted with the phenol:chloroform method, precipitated with ethanol and glycogen, and resuspended in 60 μl of 10 mM Tris–Cl, pH 8.0.

### 2.3. DNA Labelling and Hybridization

DNA labelling and hybridization was performed according to NimbleGen's protocol with our modifications.^27^ The immunoprecipitated CpG-methylated DNA (test) and the untreated, sonicated DNA (input control) were labelled by using the random priming with the Nimblegen Dual-Color DNA Labelling kit (Roche-Nimblegen, cat. no. 06370250001) with fluorescent dyes Cy3 and Cy5, respectively. The combined test and input DNA samples were suspended in hybridization buffer (Roche, cat. no. 05583683001) co-hybridized onto Mouse DNA Methylation 3 × 720 K CpG Island Plus RefSeq Promoter Arrays for 20 h at 42°C, following washing with the Nimblegen Wash Kit (Roche cat. no. 05584507001).

### 2.4. Microarray data acquisition and processing

We performed image acquisition with an MS200 Scanner (Roche, NimbleGen) at 2 µm resolution by using high-sensitivity and autogain settings. The data from scanned images were extracted and processed with DEVA v. 1.0.2 (Roche, Nimblegen) using default parameters. Data processing included obtaining log_2_ ratios, *P*-scores, and peak identification. Log_2_ ratios represent the ratios of the immunoprecipitated DNA signal to the input DNA signal. The mean log_2_ ratios were calculated using a Tukey biweight mean. *P*-scores were calculated from log_2_ ratios by performing the sliding-window (750 bp) Kolmogorov–Smirnov (KS) test around each probe applied to determine a −log10 *P*-value from the windowed KS test around that probe. The methylation peaks were obtained by merging consecutive probes with *P*-scores over the assumed cut-off (default 2.0), with maximum 500 bp spacing and minimum of two probes per peak. The processed data (*P*-scores) and raw data were deposited in the Gene Expression Omnibus (GEO) database under the accession number GSE49221.

### 2.5. Identification of differentially methylated genes

The methylation peaks were mapped to features (transcription start sites, primary transcripts, CpG islands, and other tiled regions) using DEVA v. 1.0.2, assuming the default distances 5000 bp upstream and 1000 bp downstream the feature The annotated data for the methylation peaks obtained at cut-off of 1.0 and 2.0 were merged using GenixNet, a script created by Dr P. Madanecki and adjusted to the analysed datasets, so that a single table containing all genes, tiled regions, and CpG islands with the methylation peak values for each strain and tissue at two different cut-off points was created. The data merged using GenixNet were sorted to identify the differentially methylated genes using the Excel spreadsheet. If a peak was mapped to more than one feature (e.g. transcription start site and primary transcript), the data listed in the tables of differentially methylated peaks (Supplementary Table S1) were restricted to a single feature selected according to the following order: transcription start site, primary transcript, CpG island, and tiled region.

### 2.6. Validation of the MeDIP/microarray results and gene expression level quantification

The methylation status of selected CpG nucleotides was evaluated using bisulphite sequencing and/or genomic DNA digestion with a CpG methylation-sensitive *Hpa*II restriction endonuclease. Gene expression levels were quantified using real-time PCR.

The bisulphite conversion of genomic DNA was carried out with the EZ DNA Methylation™ Kit (Zymo Research, cat. no. D5002) using 500 ng of genomic DNA. Approximately 100 ng of obtained converted DNA was used for the subsequent PCR reaction with the Maxima Hot Start Taq DNA polymerase system (ThermoScientificBio, cat. no. EP0601). The PCR reactions were performed on Veriti^®^ 96-well Thermal Cycler (Applied Biosystems). The PCR amplified DNA fragments were extracted from the agarose gel and purified using the ISOLATE Gel and PCR kit (Bioline, cat. no. BIO-52029). The PCR products were sequenced using the Applied Biosystems ABI 3730XL/ABI3700 DNA sequencer (Genomed, Poland), and the chromatograms were analysed with ContigExpress (VectorNTI 11.0, Invitrogen).

The CpG methylation-sensitive digestion of genomic DNA was carried out with the EpiJET DNA Methylation Analysis Kit based on *Msp*I/*Hpa*II digestion (ThermoScientificBio, cat. no. K1441) using 500 ng of genomic DNA and 50 ng of digested DNA for quantitative real-time PCR analysis. The methylation level was quantified using the 2-ΔCt method and expressed as the ratio between *HpaII-*digested DNA (target) and input/non-digested DNA (reference) used for enzymatic reaction. cDNA synthesis was performed with Maxima Reverse Transcriptase (ThermoScientificBio, cat. no. EP0742) using 200 ng of total RNA and oligo dT_20_. Approximately 10 ng of cDNA was used for subsequent real-time PCR reactions. The gene expression levels were calculated using the 2-ΔCt method with *Actb* as the reference gene. The results are presented as means ± SD.

Real-time PCR reactions were carried out with FastStart Essential DNA Green Master (Roche, cat. no. 06402712001) on LightCycler^®^ Nano (Roche). The primer sequences are listed in Supplementary Table S2.

### 2.7. Linear regression and scatter plots

Linear regression coefficients were determined, and scatter plots were made using the XLSTAT package (Addinsoft).

### 2.8. Chromosomal ideograms with the locations of differentially methylated genes

The chromosomal maps with the locations of differentially methylated genes were plotted using Idiographica v. 2.1 (http://www.ncrna.org/idiographica).^28^

### 2.9. Counting the DNA methylation peaks at different value ranges

The lists of unique peaks were obtained from the lists of mapped peaks generated by DEVA v. 1.0.2. The numbers of peaks at different methylation values were calculated in Excel spreadsheet.

### 2.10. Gene set enrichment analysis

The gene set enrichment analyses were performed and functional annotations were assigned by using DAVID Bioinformatics Resource 6.7 [National Institute of Allergy and Infectious Diseases (NIAID), National Institute of Health^29,30^]. An EASE Score, a modified Fisher's Exact test, was applied to calculate statistical significance of gene set enrichments.

### 2.11. Processing and analysis of genomic DNA methylation microarray data downloaded from the Gene Omnibus Expression Database

#### 2.11.1. Gene expression data

The microarray data on gene expression in the heart and liver of the MRL/MpJ mouse were retrieved from GSE19322, GSE4710, GSE25322, and GSE5241 datasets deposited in the GEO database. The gene expression values were merged with the annotations from the corresponding platform files in Excel spreadsheet. The logarithmed signal values were exponentiated. Average values were calculated from replicates. To calculate median and quartile expression values for selected gene subsets (e.g. the genes hypomethylated in the MRL/MpJ mouse), the expression values were extracted in Excel spreadsheet using gene identifiers. Gene expression values between the subsets of genes selected according to their methylation levels were compared by Wilcoxon rank-sum two-tailed tests using the SAS statistical package version 9.3 (SAS Institute, Inc., Cary, NC, USA).

#### 2.11.2. Gene methylation data

Processed DNA methylation data (*P*-scores) were downloaded from the GSE21415 dataset deposited in the GEO database. The genome coordinates were converted from the NCBI MM8 build to the NCBI MM9 build using ‘Lift Genome Annotations’ service from the University of California Santa Cruz Genome browser (http://genome.ucsc.edu/cgi-bin/hgLiftOver). The methylation peaks were obtained with NimbleScan 2.6 (Roche, Nimblegen) at cut-off of 1.0 using default parameters (at least two probes per peak with maximum spacing of 500 bp). Average values were calculated from replicates. The methylation peaks were mapped to gene features using DEVA v. 1.0.2, assuming the standard distance 5000 bp upstream and 1000 bp downstream the feature. Average values were used for the features with more than one methylation peak.

### 2.12. Analysis of correlations between DNA methylation and gene expression data

Gene expression and methylation data were merged using GenixNet. Pearson's correlation coefficients were calculated using the SAS statistical package version 9.3 (SAS Institute, Inc.).

### 2.13. Analysis of correlations between genome-wide DNA methylation profiles

As the formats of microarray platforms applied in this study and that by Liang *et al*. are different, we restricted the analysis to the peaks mapped to the transcription start sites shared by both platforms (18 199 unique genes included in Roche MM8 385 K RefSeq Promoter Array). If more than a single peak was mapped to a gene, average values were calculated by using Excel ‘pivot tables’ function. The methylation peaks for this analysis were obtained at cut-off of 1.0 in order to reduce the number of ‘0’ under the cut-off, which, in fact, represent a spectrum of values. The peaks under the cut-off of 1.0 were given the value ‘0’. The arrays were merged using Entrez identifiers. Pearson's correlation coefficients were calculated using the SAS statistical package version 9.3 (SAS Institute, Inc.).

## 3. Results and discussion

We investigated the epigenetic basis of the regeneration phenomenon in the MRL/MpJ mouse using genome-wide DNA methylation profiling. As the MRL/MpJ strain does not have an immediate relative, which does not exhibit enhanced regeneration abilities, we chose the C57BL/6J mouse as the reference strain. We carried out genome-wide DNA methylation profiling in hearts, livers, and spleens of the MRL/MpJ and C57BL/6J strains using the MeDIP approach followed by microarray analysis. The Nimblegen microarray system ‘3 × 720 K CpG Island Plus RefSeq Promoter’ we applied in the study includes 20 404 promoter regions; 22 881 transcripts and 15 980 CpG Islands. The DNA methylation profiles we obtained for the MRL/MpJ and C57BL/6J were compared in order to identify the differentially methylated genes and regions. The overall comparisons of inter-strain similarities and differences between the examined genomic DNA methylation profiles are visualized using scatter plots (Supplementary Fig. S1).

This study was designed as an initial analysis of genome-wide DNA methylation patterns and we restricted the selection of tissues to heart, liver, and spleen. Heart and liver were selected to contrast the non-regenerating organ with the regenerating one. Spleen was chosen for its role in the immune system, as immune response to injury seems to be one of the most critical events in the regeneration process.^31^

### 3.1. Genes differentially methylated in the MRL/MpJ mouse

As the DNA methylation patterns are prone to individual and conditional variation, we focused on the analysis of the genes revealing the most distinguishing differences in the methylation status. We assumed a gene/region to be differentially methylated if a methylation peak mapped to this gene/region meets at least one of two conditions: either the peak value is at least 3.0 or higher and it is at least twice higher than in the reference (at cut-off of 1.0) or it is at least 2.0 and it is lower than 1.0 for the reference (see Section 2 for the definition of peak value). A peak is mapped to a feature (transcription start site, primary transcript, CpG island, or tiled region) if it is located within 5000 bp upstream and 1000 bp downstream the region (a transcription start site, primary transcript, or CpG island).

**Figure 1.**
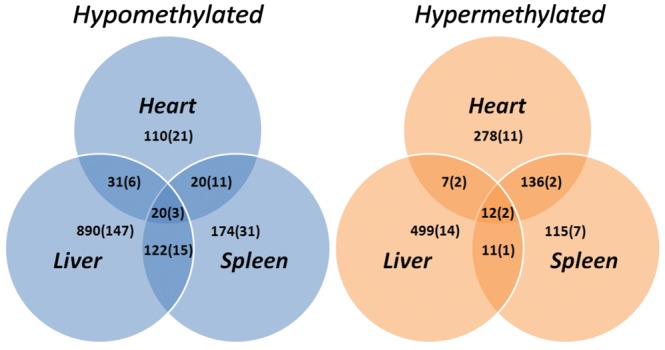
The numbers of differentially methylated genes and CpG islands in the MRL/MpJ mouse in the heart, liver, and spleen tissues. The numbers of CpGs distant from the genes (out of −5000 bp and + 1000 bp range) in brackets. It is assumed here that a gene/region is differentially methylated if it meets at least one of two conditions: either (i) the peak value is at least 3.0 or higher and it is at least twice higher than in the reference or (ii) it is at least 2.0 and it is lower than 1.0 for the reference.

**Figure 2.**
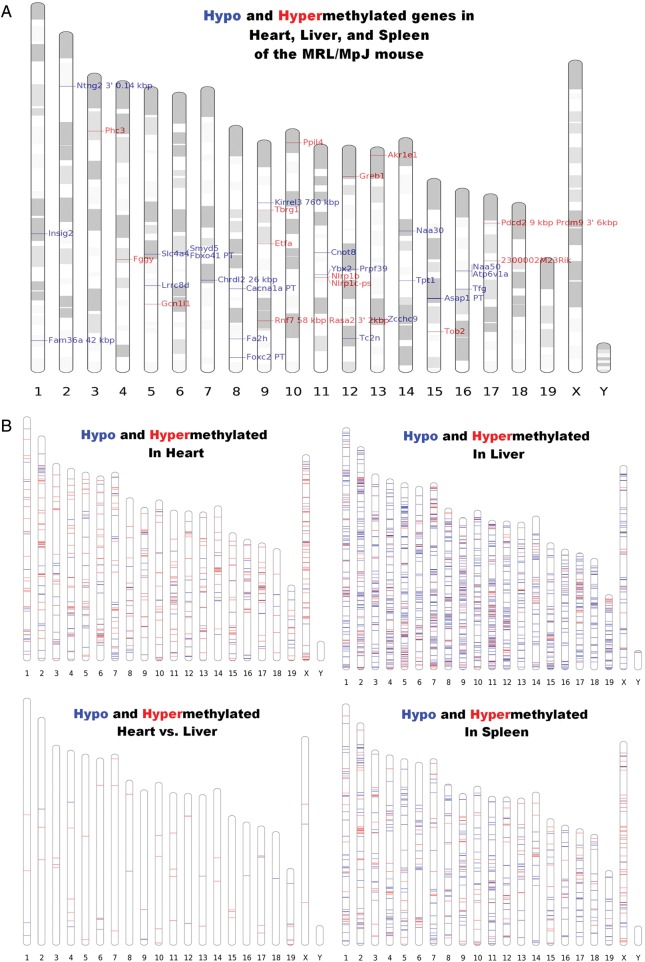
The chromosomal locations of genes and CpG islands differentially methylated in the MRL/MpJ mouse. (A) The genes and CpG islands that are differentially methylated in the three examined tissues: heart, liver, and spleen. PT - DNA methylation peak mapped to a primary transcript, kb—distance in kb is given if the methylation peak is located out of the range 5000 bp upstream and 1000 bp downstream the feature. (B) The genes and CpG islands differentially methylated in the heart, liver, and spleen. The genes displaying substantially higher and lower methylation degree in the MRL/MpJ mouse are marked red (hypermethylated, grey in the print) and blue (hypomethylated, black in the print), respectively. The values and genomic coordinates of the DNA methylation peaks as well as the descriptions of mapped of genes are listed in Supplementary Table S1. Heart vs. liver analysis singled out the genes and CpG islands, which have different methylation status in the heart than in the liver of the MRL/MpJ, but not the C57BL/6J, mouse. (see the chapter: ‘Genes with similar DNA methylation status in the heart and liver of the MRL/MpJ but not the C57BL/6J mouse’).

As the result several hundreds of differentially methylated genes, we will refer to as ‘hypomethylated’ and ‘hypermethylated’, were identified in liver, spleen, and heart tissue of the MRL/MpJ mouse (Fig. 1 and Supplementary Table S1). The majority of differentially methylated genes and regions were not shared by the three examined tissues: heart, liver, and spleen. Twenty-three genes and CpG islands were found to be hypomethylated and 14 to be hypermethylated in the MRL/MpJ mouse in the three examined tissues. These genes and CpG islands were located on all chromosomes except the 18, 19, X, and Y (Fig. 2), and they are associated with a number of different functions. The complete list and the descriptions of these genes are presented in Supplementary Table S1, while a selection of remarkable representatives of this group (Table 1) are discussed later in this article.

**Table 1.** The remarkable representatives of the genes differentially methylated in the MRL/MpJ mouse

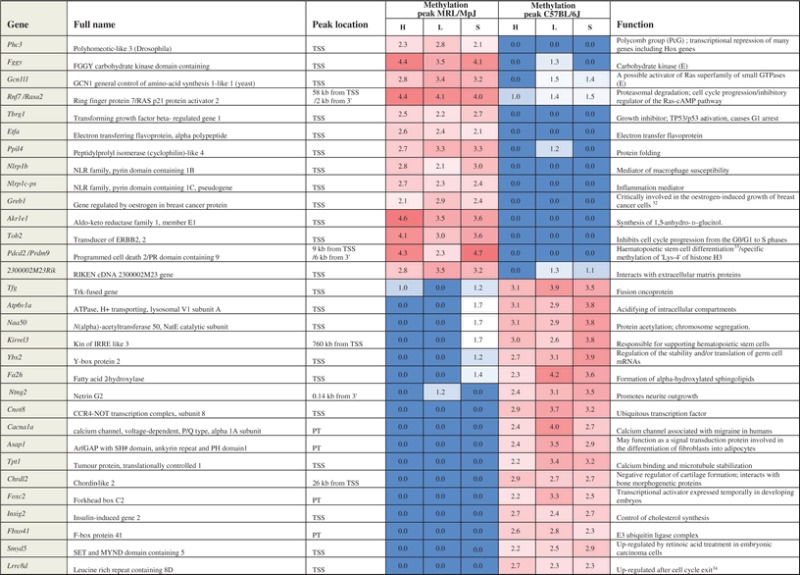
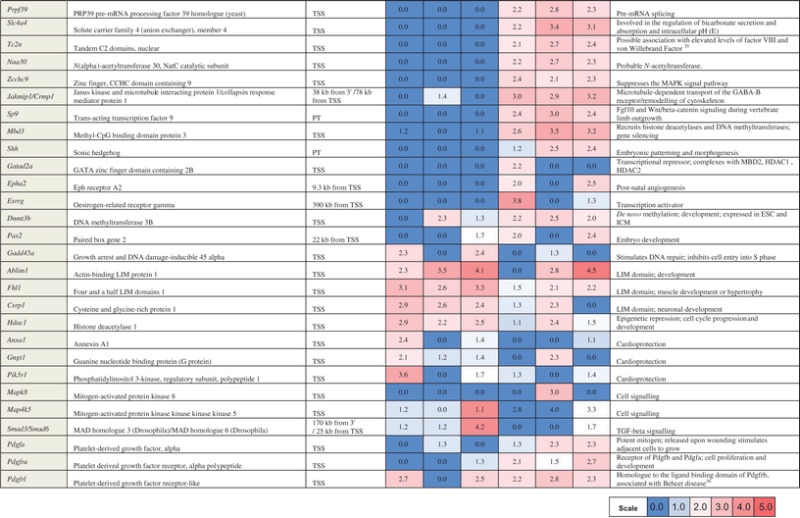

If not indicated otherwise, the information on gene functions is taken from DAVID and Gene Entrez (E, http://www.ncbi.nlm.nih.gov/gene/).

H: heart; L: liver; S: spleen; TSS: transcription start site; PT: primary transcript.

### 3.2. Validation of the MeDIP/microarray results

A selection of DNA methylation peaks were validated in the MRL/MpJ and the C57BL/6J reference using bisulphite sequencing and/or digestion with a CpG methylation-sensitive restriction endonuclease *Hpa*II, followed by quantitative real-time PCR analysis [Supplementary Table S3 (summarized results), Supplementary File S1 (experiment details), and Supplementary File S2 (representative DNA sequencing chromatograms assembled with the reference sequences)]. Gene expression levels of selected genes were examined by quantitative real-time PCR analysis [Supplementary Table S3 (summarized results) and Supplementary File S1 (experiment details)].

### 3.3. The chromosomal locations of differentially methylated regions

The hyper- and hypomethylated genes and regions were found on all chromosomes. Several chromosomal loci display significant positional enrichments of the genes differentially methylated in the MRL/MpJ mouse, but no remarkable regions of peak accumulation could be distinguished (Fig. 2A and B).

### 3.4. Gene methylation status and gene expression

**Figure 3.**
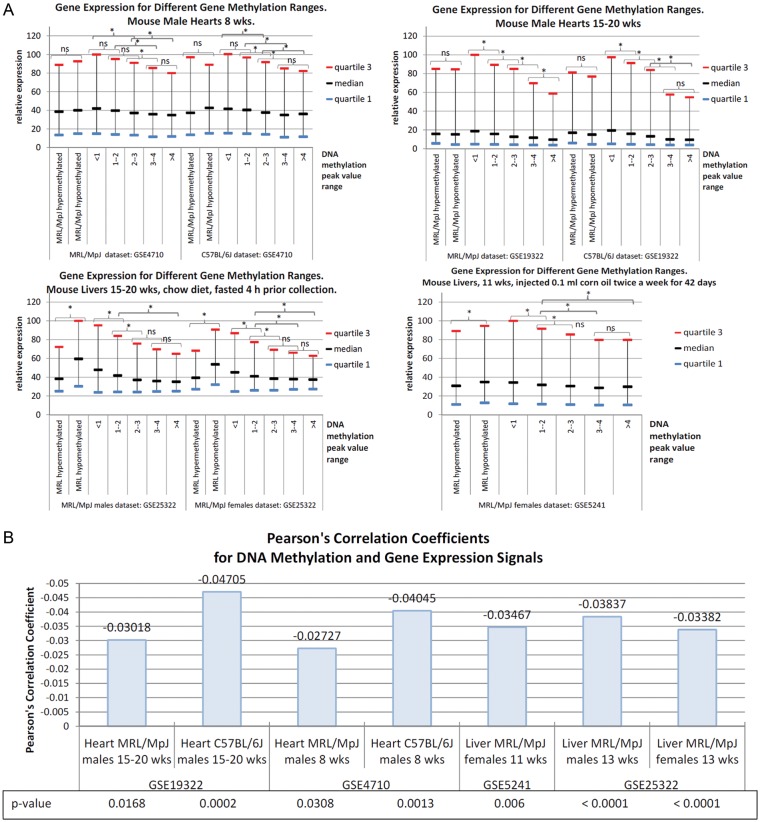
Gene methylation and gene expression profiles. (A) Gene expression levels at different DNA methylation peak value ranges in heart and livers of the MRL/MpJ mouse. The microarray gene expression data representing the tissues collected from uninjured organs were retrieved from the following datasets GSE4710 and GSE19222 (hearts), GSE5241 and GSE25322 (livers) that had been obtained in four independent studies.^16,17,37,38^ Wilcoxon rank-sum two-tailed tests were performed in order to compare gene expression values between the groups of genes selected according to the DNA methylation level ranges (determined in this study). The median and quartile values were normalized to the highest value from each dataset. (B) The correlations between the DNA methylation and gene expression levels. The Pearson's correlation coefficients were calculated for the gene methylation values determined in this study for the heart and liver of the MRL/MpJ and C57BL/6J mouse, and corresponding gene expression values that were obtained from four other independent studies,^16,17,37,38^ as described above.

DNA methylation of gene promoters is known to repress gene expression. With regard to evaluating the impact of DNA methylation on gene expression in the examined tissues, we analysed the available expression microarray data for the MRL/MpJ heart and liver tissues reported in four different studies.^16,17,37,38^ Our evaluation was based on the correlations between DNA methylation status and expression signals, as well as mean values of expression signals calculated for subsets of genes at different peak value ranges. We found that the expression signal values were increasing with the decrease of DNA methylation status (Fig. 3A), and the values of methylation peaks and gene expression signals were reversely correlated (Fig. 3B). The Pearson's correlation coefficients for gene methylation and expression values in the heart though significant for both the C57BL/6J and MRL/MpJ mouse were lower for the latter one. This analysis confirmed that the obtained genomic DNA methylation patterns are adequate to the transcriptomic profiles. However, the gene expression signals in hearts for the groups of genes differentially methylated in the MRL/MpJ and C57BL/6J did not show statistically significant differences between these two strains (Fig. 3A). This inconsistence could be explained by age differences, as we used DNA methylation data obtained for 5-week-old animals and the gene expression data had been collected for much older mice (8- to 20-week old). It should also be noted that the majority of DNA methylation peaks mapped to the differentially methylated genes in the MRL/MpJ are located at higher distance than those in the C57BL/6J mouse (Fig. 6B). After all, in spite of the age differences, the overall correlations between DNA methylation and gene expression were confirmed.

We also would like to punctuate three possible reasons why gene methylation and expression levels could be inconsistent. First, it should be noted that tissues unlike cell lines are more likely to be mosaics of cells that show different DNA methylation and gene expression patterns, thus a methylation peak may represent gene methylation status in a fraction of tissue cells, while another fraction of cells is responsible for high gene expression. Secondly, an individual gene does not have to show higher expression when unmethylated, as other repression mechanisms could be involved. Thirdly, some genes are induced due to demethylation of selected CpGs within a CpG island.^39^

### 3.5. Functional profiling of genes differentially methylated in the MRL/MpJ mouse: gene set enrichment analysis

Once the genes differentially methylated in the MRL/MpJ mouse were singled out, we could examine which functional and structural categories were overrepresented among these genes, to be followed by looking for associations with the phenotype. As the first step, we carried out gene set enrichment analysis for the groups of hypo- and hypermethylated genes using the DAVID algorithm and database.^29,30^ We examined the sets of genes hypo- and hypermethylated in hearts, livers, and spleens (six gene sets, Supplementary Table S1). The analysis revealed a number of statistically significant functional terms (Supplementary Table S4). Although terms which are not statistically significant, as well as those found for one of tissues, could provide useful clues on the association between DNA methylation and regeneration potential, we decided to focus on the statistically significant terms representative of the three examined tissues. The statistically significant functional terms representing the hypomethylated genes in the three examined tissues (Supplementary Table S5) could be associated with three major categories: phosphoproteins, alternative splicing, and negative regulation of metabolism (repressors), thus suggesting that the MRL/MpJ mouse is likely to produce more phosphoproteins, repressors, and alternatively spliced transcripts. Interestingly, intragenic DNA methylation has been associated with the use of alternative promoters and the synthesis of alternative transcripts,^40^ while positive regulation of phosphate metabolic processes in human foetal heart tissues has been concluded from the analysis of DNA methylation microarray data.^41^

An additional term shared by the term sets obtained for the genes hypomethylated in liver, heart, and spleen of the MRL/MpJ mouse was associated with embryonic morphogenesis. This suggests that the DNA methylation pattern of MRL/MpJ mouse might have retained some embryonic features and the possibility will be discussed in the next chapter.

The remarkable features of the functional terms representing the hypermethylated genes identified in the heart and spleen of the MRL/MpJ mouse were associated with three groups: G-protein-coupled receptors, including mostly olfactory receptors, Ig-like V-type, and natural killer receptor Ly49 genes. Over 1500 olfactory receptor genes are one of the most abundant gene family in mouse, and this may explain their overrepresentation. Nevertheless, it is worth noting that altered olfactory function has been reported in the MRL mouse model of the central nervous system lupus.^42^ The majority of selected Ig-like V-type genes belongs to the cluster of selection and upkeep of intraepithelial T cell genes. The region of chromosome 4, where the selection and upkeep of intraepithelial T cells genes are located, is polymorphic, and we found that the MRL/MpJ mouse lacks the nucleotide sequences corresponding to the DNA methylation peaks in the promoter regions of *Skint3* and *Skint4* (not shown), so it is not the epigenetic but the genetic impact rather which should be considered for this group of genes. The hypermethylated genes of natural killer Ly49 receptors included mostly killer cell lectin-like receptor subfamily A genes: *Klra12*, *Klra13-ps*, *Klra4*, *Klra15*, *Klra23*, *Klra22*, *Klra18*, and *Klra33*, located on the chromosome 6.

None of the functional terms produced for the genes hypermethylated in the liver was shared by the term sets produced for the genes hypermethylated in the heart and spleen of the MRL/MpJ mouse.

### 3.6. Do the genomic DNA methylation patterns of the MRL/MpJ mouse retain embryonic features?

Enhanced healing typical of foetal and neonatal period suggests that the regeneration abilities of the MRL/MpJ mouse could be embryonic relics preserved in the adult organism. As indicated by Naviaux *et al.*,^24^ the adult *MRL*/*MpJ* mouse retains a selection of embryonic metabolic features. With regard to finding out as to whether the genomic DNA methylation profiles of the MRL/MpJ mouse exhibit embryonic traits, we investigated the representation of the genes associated with embryo development among the genes differentially methylated in the MRL/MpJ mouse, as well as we evaluated the overall similarity of the MRL/MpJ and embryonic methylation patterns.

### 3.7. Genes associated with embryonic development are overrepresented among those hypomethylated in the MRL/MpJ mouse

The gene set enrichment analysis for the genes hypomethylated in the MRL/MpJ showed excessive numbers of genes associated with embryonic development and homeobox genes (Table 2). ‘Embryonic organ morphogenesis,’ one of the terms related to embryonic development, was shared by the functional term sets produced for the three examined tissues. Three different groups of genes associated with embryonic organ morphogenesis which were identified in the liver, heart, and spleen shared two elements: the insulin induced gene 2 (*Insig2*) and forkhead box C2 (*Foxc2*) (Supplementary Table S6). The latter could be of particular interest of regenerative medicine as a potent regulator of angiogenesis.^43^

**Table 2.** Excessive numbers of homeobox and embryonic morphogenesis genes are hypomethylated in the tissues of adult MRL/MpJ mouse

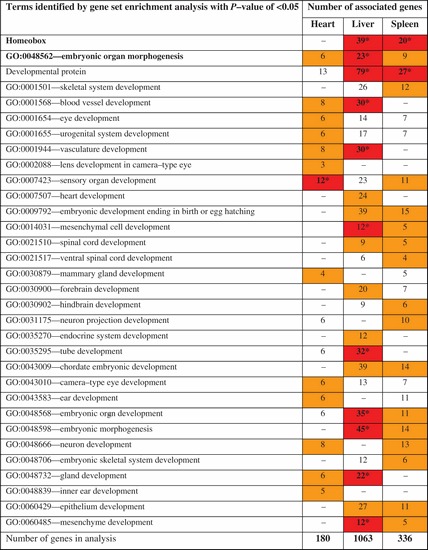

The gene set enrichment analysis carried out using DAVID showed that homeobox genes and a number of other genes involved in multiple developmental processes were significantly overrepresented in the group of the MRL/MpJ hypomethylated genes (Supplementary Table S1). The enrichment terms which are statistically significant with modified Fisher's exact test (*p*-values <0.05) are indicated with orange fields (darker shade of grey in the print) and those with *p*-values <0.05 after Benjamini correction are indicated with red fields (darker shade of grey in the print).

Homeobox genes have been found to be hypomethylated in the embryos of the C57BL/6J mouse in hearts and livers (spleens were not examined in that study).^44^ Homeobox genes were overrepresented among the genes differentially methylated in the spleens and livers, but not in the hearts of the MRL/MpJ mouse. Thirty-nine and 20 homeobox genes were found to be hypomethylated in the liver and spleen in the MRL/MpJ mouse, respectively (Table 2 and Supplementary Table S6). Seven of these homeobox genes were hypomethylated in both tissues.

### 3.8. Genomic DNA methylation patterns of the MRL/MpJ mouse reveal lower inter-tissue diversification

**Figure 4.**
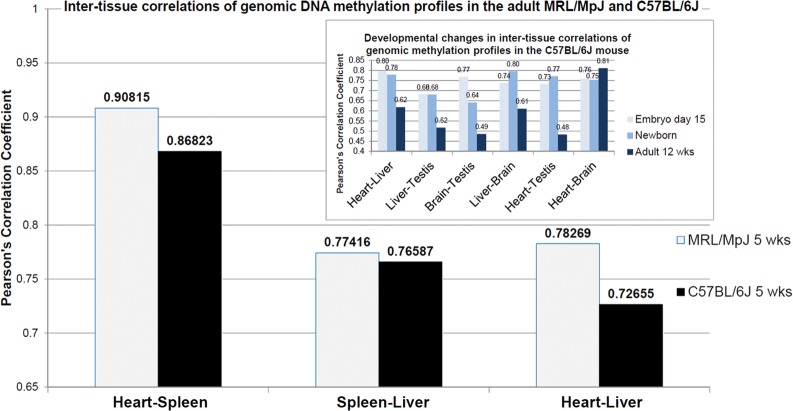
Genome-wide DNA methylation profiles display lower inter-tissue diversification in the MRL/MpJ than in the C57BL/6J mouse. Inset: Similarly, we observed lower inter-tissue diversification of genome-wide DNA methylation profiles reported by Liang *et al.*^44^ in embryos and newborns than in adults (with the exception for the correlations between the heart and brain). The inter-tissue correlations between pairs of genomic DNA methylation profiles were determined using Pearson's correlations coefficients. All Pearson's correlation coefficients were statistically significant with *P*-values of <0.0001. Pearson's correlation coefficients were calculated for the pairs of genomic DNA methylation arrays obtained in this study: heart–spleen, spleen–liver, and heart–liver for both the MRL/MpJ and C57BL/6J mouse. Analogically, Pearson's correlation coefficients were determined for the genomic DNA methylation profiles in different tissues of murine embryos, newborns, and adults reported by Liang *et al.*^44^ The microarray data were extracted from the dataset deposited in the GEO database under the accession number GSE21415. The Pearson's correlation coefficients shown in the inset were determined for the average values obtained from two biological replicates, but similar trends were found in the correlations determined for individual animals (not shown).

To evaluate the overall similarity between the genomic DNA methylation profiles of embryos and the adult MRL/MpJ mouse, we applied Pearson's correlation test. We analysed the DNA methylation data obtained in our study and that reported by Liang *et al.*^44^ As we were comparing the data produced by different generations of Nimblegen microarrays, we restricted the analysis to the transcription start sites shared by both platforms. The analysis of correlation data showed that the inter-tissue diversification of the genomic DNA methylation profiles was lower in the MRL/MpJ than in the C57BL/6J mouse (Fig. 4). Assuming that the DNA methylation patterns reflect tissue differentiation, which advances with development, the lower diversification of inter-tissue DNA methylation patterns seems likely to occur at earlier developmental stages. With regard to this hypothesis, we analysed the changes in correlation coefficients between the genomic DNA methylation profiles in embryo, newborn, and adult tissues using the data reported by Liang *et al.*^44^ to find out that in most cases the inter-tissue correlations of DNA methylation profiles decrease with age (Fig. 4, inset). To our knowledge, low inter-tissue diversification of the embryonic DNA methylation profiles has never been reported to date.

The genomic DNA methylation profiles of the adult MRL/MpJ mouse are not embryonic, but they display several features resembling those of embryos. The analysis of genomic DNA methylation patterns in the embryonic and newborn tissues of the MRL/MpJ mouse is necessary to provide further evidence.

### 3.9. Enhanced proportion of high methylation peaks in the MRL/MpJ compared with C57BL/6J mouse

**Figure 5.**
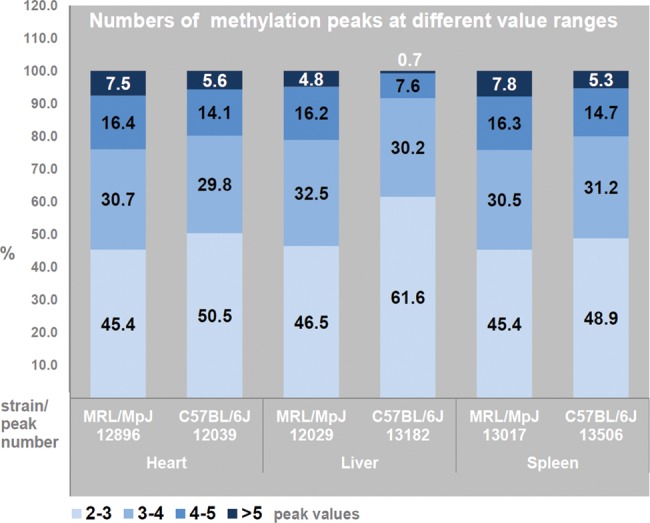
Excessive numbers of high DNA methylation peaks in the genome-wide DNA methylation profiles in the tissues of MRL/MpJ mouse. It is assumed that high methylation peaks have the values of 4.0 and over.

In addition to the above-discussed low inter-tissue diversification, another characteristic feature of the genomic DNA methylation profiles of the MRL/MpJ mouse, we found, is an enhanced proportion of high methylation peaks (with the values of 4.0 and over) in comparison with the C57BL/6J (Fig. 5).

### 3.10. The balance of DNA methylation is shifted upstream the genes in the MRL/MpJ mouse

It is not only the degree of CpG methylation in the regulatory region of a gene, but also the distance of methylated CpGs from the transcription start sites that contributes to the repression of gene expression.^39,45^ In other words, a DNA methylation peak though mapped to a gene may have different impacts on gene expression dependent on its location.

**Figure 6.**
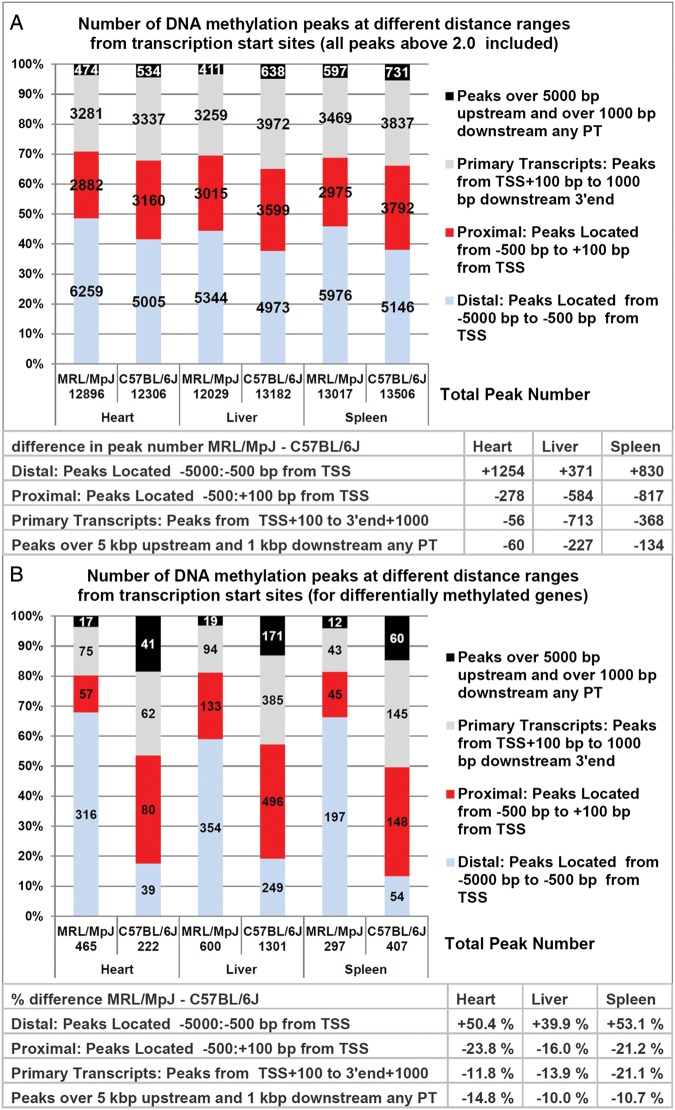
The numbers of DNA methylation peaks at different distance range from transcription start sites and primary transcripts in the MRL/MpJ and C57BL/6J mouse. DNA methylation peaks were divided into four categories, according to their location from the genes they were mapped to: (i) distal, mapped from 5000 bp upstream up to 500 bp upstream from transcription start sites (TSSs), (ii) proximal, mapped from 500 bp upstream up to 100 bp downstream from transcription start sites, (iii) mapped within primary transcripts (PT) and 1000 bp downstream from PT 3′ ends, except those up to 100 bp from TSSs, (iv) the peaks within remote CpG islands that were not mapped to any PT, i.e. those located over 5000 bp upstream from TSSs and 1000 bp downstream from PT 3′ ends. (A) Peak counts including all DNA methylation peaks of ≥2.0. (B) Peak counts including differentially methylated peaks only (the peaks selected according to the criteria specified in the chapter: ‘Genes differentially methylated in the MRL/MpJ mouse’).

To examine the distribution of DNA methylation peaks in relation to the distance from their promoters, we calculated peak numbers at different distance ranges from transcription start sites and primary transcripts in hearts, livers, and spleens of the MRL/MpJ and C57BL/6J mouse (Fig. 6). The microarray design of the Nimblegen platform we used includes mainly the transcription start sites associated with the basal promoters.

We found that the number of DNA methylation peaks situated over 500 bp upstream from transcription start sites in the MRL/MpJ substantially exceeds than that in the C57BL/6J mouse (Fig. 6A). The number of DNA methylation peaks mapped within close promoter neighbourhood, in the range from 500 bp upstream up to 100 bp downstream from transcription start sites, is in turn higher in the C57BL/6J than that in the MRL/MpJ mouse (Fig. 6A). Such differences were revealed in all examined tissues and they do not reflect the differences in total peak numbers. The numbers of DNA methylation peaks located within the primary transcripts and up to 1000 bp downstream, which are mostly the intragenic ones, were remarkably higher in the C57BL/6J than that in the MRL/MpJ mouse in the spleens and livers, while in the hearts they were similar (Fig. 6A).

We also found that the numbers of DNA methylation peaks mapped over 5000 bp upstream and over 1000 bp downstream from primary transcripts, we may consider intergenic, are lower in the MRL/MpJ compared with the C57BL/6J mouse (Fig. 6A). However, the microarray platform, we used, is a promoter one, and few probes are derived from the intergenic regions. Consequently, this estimate of intergenic DNA methylation may not be representative.

Interestingly, the differences described above are dramatically more pronounced for the differentially methylated genes (Fig. 6B).

The results indicate that the balance of DNA methylation in the MRL/MpJ mouse is shifted towards the distal alternative promoters and other distal regulatory elements as, for example, enhancers. This shift is particularly remarkable for the differentially methylated genes. However, it should be stressed that the observed methylation of distal regulatory regions is not prevailing, but rather more accentuated in the MRL/MpJ than in the C57BL/6J mouse. Alternative promoters are known to be associated with development and tissue and cell differentiation,^46,47^ which implicates the question as to whether this trait of DNA methylation profiles could impact the cellular differentiation potential in the MRL/MpJ mouse.

### 3.11. Genes with similar DNA methylation status in the heart and liver of the MRL/MpJ, but not the C57BL/6J, mouse

As regeneration abilities of mammalian liver remarkably exceed those of the heart, the genes that display strong differences in the DNA methylation status between heart and liver could be of particular interest with regard to regeneration. Therefore, we singled out the genes that had ‘liver-like’ methylation status in the MRL/MpJ, but not in the C57BL/6J heart. We identified two, relatively small, groups of such genes. One group consisting of 18 genes and CpG islands were the genes that were hypermethylated in the heart and hypomethylated in the C57BL/6J liver, while hypomethylated both in the heart and liver of the MRL/MpJ mouse. Three genes involved in embryonic morphogenesis: *Epha2*, *Pax2*, and *Gatad2a* (Table 1) deserve particular attention in this group. The other set, including 47 genes and CpG islands, were hypermethylated in the C57BL/6J heart and hypomethylated in the liver, while hypermethylated both in the heart and liver of the MRL/MpJ mouse. Two groups of genes, 10 genes associated with acetylation, and 3 genes encoding LIM domains were overrepresented in this group (Supplementary Table S4). The result was not statistically significant for the first group, but it includes as an important gene as histone deacetylase 1 (*Hdac1*), which is one of key factors responsible for chromatin remodelling. The repression of this factor could prevent chromatin condensation, thus promoting the transcription of a number of target genes. The overrepresentation of three LIM zinc-binding domain genes (*Csrp1*, *Ablim1*, and *Fhl1*) was statistically significant, and LIM domains are known to play important roles in the regulation of developmental pathways.^48^

### 3.12. Remarkable representatives of the differentially methylated genes

As we identified as many as hundreds of differentially methylated genes in three tissues of the MRL/MpJ mouse (Supplementary Table S1), we would like to give a short description of a selection of these genes (Table 1). The selection includes the genes differentially methylated in all examined tissues and several others that could be of exceptional interest due to their potential importance to regenerative abilities. A few genes from this list which arrested our distinct attention and were not discussed before are discussed below.

Since the MRL/MpJ mouse exhibits enhanced healing abilities, wound repair-related genes could be expected to be found in the group of the genes differentially methylated in this strain. As the genes related to wound healing are not a precisely defined category, it is difficult to evaluate if such genes are overrepresented among those differentially methylated in the MRL/MpJ. We found two genes associated with wound healing among the genes hypomethylated in the MRL/MpJ mouse that deserve particular attention: platelet-derived growth factor alpha (*Pdgfa*) and platelet-derived growth factor receptor alpha (*Pdgfra*). The roles of platelet-derived growth factors (platelet-derived growth factor beta in particular) and their receptors in wound healing have already been established.^49^

The promoter regions of the gene encoding *de novo* methylase Dnmt3b are hypomethylated in the heart and spleen of the MRL/MpJ mouse. Dnmt3b could be of particular importance as it is expressed in embryonic stem cells and embryo inner cell mass and when expressed in adults could contribute to epigenetic plasticity.

The promoter regions of the growth arrest and DNA damage-inducible 45 alpha (*Gadd45a*) gene were hypermethylated in the MRL/MpJ mouse in the spleen and heart (but not in the liver). Interestingly, enhanced methylation of this region has been observed in response to folate intervention during spinal cord regeneration.^2^

Regeneration abilities are often associated with the pluripotency of stem cells. Nevertheless, the list of genes differentially methylated in the MRL/MpJ mouse does not include the key stem cell marker genes, such as *Oct4* (*Pouf51*), *Klf4*, *Sox2*, *Nanog*, and *Islet1* (which are covered by the array), where the three latter have been reported to display enhanced expression in the MRL/MpJ mouse.^24^

## 4. Concluding remarks

The analysis of genomic DNA methylation profiles revealed hundreds of genes hypo- and hypermethylated in the heart, spleen, and liver of the MRL/MpJ mouse strain in comparison with the C57BL/6J control, and 37 of these genes were differentially methylated in the three examined tissues. The distinguishing features of the genomic methylation patterns in the tissues of adult MRL/MpJ mouse, we found, were the shift of DNA methylation balance upstream the genes and relatively low inter-tissue diversity, resembling that we observed in previously reported genomic DNA methylation profiles of embryos and newborns.^44^ The possibility that the DNA methylation patterns of the MRL/MpJ mouse retain embryonic relics is supported by another observation, which is the overrepresentation of the genes involved in embryogenesis among the genes hypomethylated in the MRL/MpJ mouse. To our knowledge, this is the first study to report embryonic features of the genomic DNA methylation profile in adult organism.

This study presents a group of genes differentially methylated in the MRL/MpJ mouse, which could be analysed as candidate genes of regenerative phenotype. The examination of additional reference strains is necessary to narrow the group by eliminating the genes related to inter-strain differences that are not linked with regeneration. Our results show that the embryo-like features of the DNA methylation patterns are likely to contribute to enhanced regenerative capacity observed in different tissues of the MRL/MpJ mouse. The analysis of the DNA methylation changes in the wound area in the MRL/MpJ and non-healing reference strains is necessary to examine the epigenetic differences which occur in response to injury.

The *Fas*^lpr^ mutant of the MRL/MpJ mouse has been widely investigated as a model of systemic lupus erythematosus and other autoimmune diseases. The MRL/MpJ mouse has been also examined in the context of cholesterol metabolism.^38^ Although we focused on wound healing and regeneration aspects rather, we think that our data could also be useful for autoimmune and lipid metabolism studies.

## Supplementary Data

Supplementary data are available at www.dnaresearch.oxfordjournals.org.

## Funding

This study was supported by the research grant of National Science Centre of Poland 2011/01/B/NZ2/05352 and in part by the Foundation for Polish Science (FNP) support grant for the Kolumb programme fellows ‘The molecular basis of regeneration in the MRL mouse’ awarded to Dr P. Sachadyn.

## Supplementary Material

Supplementary Data
