# Supplementary material for: Epigenetic Basis of Regeneration: Analysis of Genomic DNA Methylation Profiles in the MRL/MpJ Mouse
Source: DNA Res. 2013 Aug 8;20(6):605–21. doi: 10.1093/dnares/dst034 (PMC3859327; doi:10.1093/dnares/dst034)
Supplement: Supplementary Data [file supp_20_6_605__index.html]

Epigenetic Basis of Regeneration: Analysis of Genomic DNA Methylation Profiles in the MRL/MpJ Mouse — Supplementary Data 

# Epigenetic Basis of Regeneration: Analysis of Genomic DNA Methylation Profiles in the MRL/MpJ Mouse

## Supplementary Data

Supplementary Data

**Files in this Data Supplement:**

- Supplementary Data - zip file
- Supplementary File - xlsx file
- Supplementary Table 1 - xlsx file
- Supplementary Table 2 - doc file
- Supplementary Table 3 - doc file
- Supplementary Table 4 - xlsx file
- Supplementary Table 5 - doc file
- Supplementary Table 6 - doc file
