# Supplementary material for: Epigenetic Basis of Regeneration: Analysis of Genomic DNA Methylation Profiles in the MRL/MpJ Mouse
Source: DNA Res. 2013 Aug 8;20(6):605–21. doi: 10.1093/dnares/dst034 (PMC3859327; doi:10.1093/dnares/dst034)
Supplement: Supplementary Data [file supp_dst034_dst034supp_data.zip › bAkr1e1.pdf]

Section 1

40 50 69

» Reference-bA... (38) T A A T T C G T T T G G G G A A A A T T T T A A T T A G A
» BHt6-bAkr1e1... (38) T A A T T T G T T T G G G G A A A A T T T T A A T T A G A
T A A T T T G T T T G G G G A A A A T T T T A A T T A G A

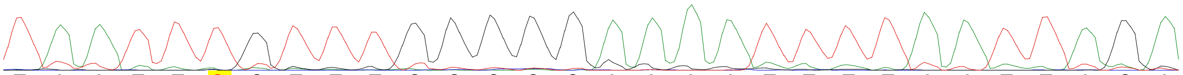

» MHt5-bAkr1e... (40) T A A T T C G T T T G G G G A A A A T T T T A A T T A G A
T A A T T C G T T T G G G G A A A A T T T T A A T T A G A

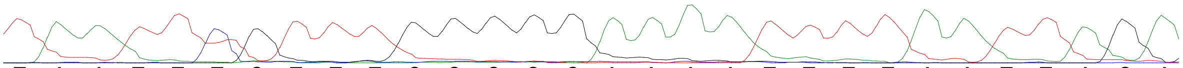

Contig 2 (40) T A A T T T G T T T G G G G A A A A T T T T A A T T A G A
+

Section 2

70 80 99

» Reference-bA... (68) G T T C G G G T T T G G A A G G A A T T A T T T T A G G T A
» BHt6-bAkr1e1... (68) G T T T G G G T T T G G A A G G A A T T A T T T T A G G T A
G T T T G G G T T T G G A A G G A A T T A T T T T A G G T A

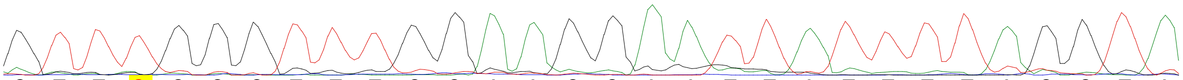

» MHt5-bAkr1e... (70) G T T C G G G T T T G G A A G G A A T T A T T T T A G G T A
G T T C G G G T T T G G A A G G A A T T A T T T T A G G T A

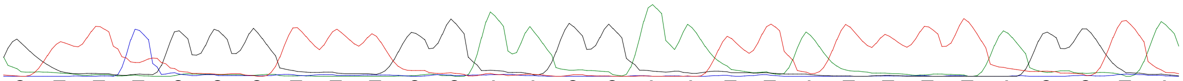

Contig 2 (70) G T T T G G G T T T G G A A G G A A T T A T T T T A G G T A
+

Section 3

100 110 129

» Reference-bA... (98) A A A G A A A T T T C G T G T T T T T G G T T G T T T T T G
» BHt6-bAkr1e1... (98) A A A G A A A T T T T G T G T T T T T G G T T G T T T T T G
A A A G A A A T T T T G T G T T T T T G G T T G T T T T T G

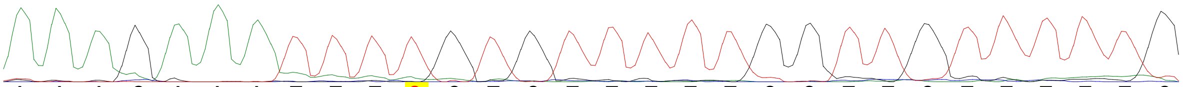

» MHt5-bAkr1e... (100) A A A G A A A T T T C G T G T T T T T G G T T G T T T T T G
A A A G A A A T T T C G T G T T T T T G G T T G T T T T T G

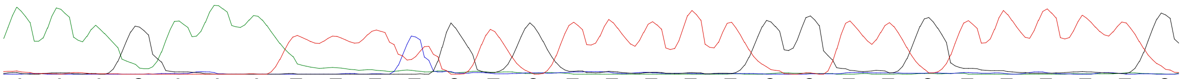

Contig 2 (100) A A A G A A A T T T T G T G T T T T T G G T T G T T T T T G
+

Section 4

130 140 159

» Reference-bA... (128) A G T A T G T A T T T A T T A G G T T T C G G G A T T T T T
» BHt6-bAkr1e1... (128) A G T A T G T A T T T A T T A G G T T T T G G G A T T T T T
A G T A T G T A T T T A T T A G G T T T T G G G A T T T T T

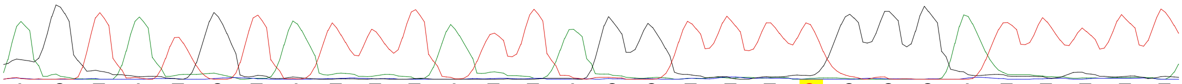

» MHt5-bAkr1e... (130) A G T A T G T A T T T A T T A G G T T T C G G G A T T T T T
A G T A T G T A T T T A T T A G G T T T C G G G A T T T T T

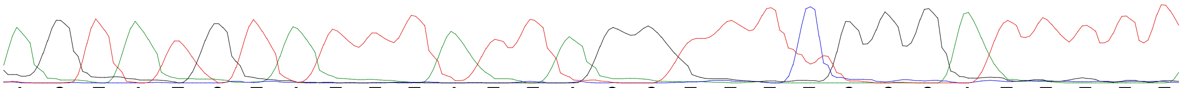

Contig 2 (130) A G T A T G T A T T T A T T A G G T T T T G G G A T T T T T
+

Section 5

160 170 189

» Reference-bA... (158) A G T T T T A T T T A T A T C G G A G T
» BHt6-bAkr1e1... (158) A G T T T T A T T T A T A T T G G A G T
A G T T T T A T T T A T A T T G G A G T

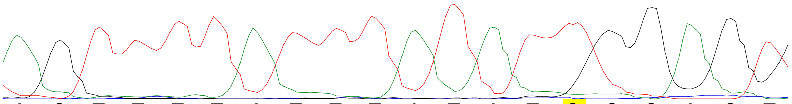

» MHt5-bAkr1e... (160) A G T T T T A T T T A T A T C G G A G T
A G T T T T A T T T A T A T C G G A G T

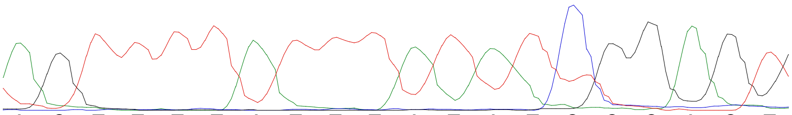

Contig 2 (160) A G T T T T A T T T A T A T C G G A G T
+
