# Supplementary material for: Epigenetic Basis of Regeneration: Analysis of Genomic DNA Methylation Profiles in the MRL/MpJ Mouse
Source: DNA Res. 2013 Aug 8;20(6):605–21. doi: 10.1093/dnares/dst034 (PMC3859327; doi:10.1093/dnares/dst034)
Supplement: Supplementary Data [file supp_dst034_dst034supp_data.zip › bNanog.pdf]

Section 1

52 60 70 81

» Reference-bN... (46) T G G G G C G T G G G T G T C G T T T G G G T G T T T G G G

» BLiv4-bNanog... (46) T G G G G C G T G G G T G T C G T T T G G G T G T T T G G G

T G G G G C G T G G G T G T C G T T T G G G T G T T T G G G

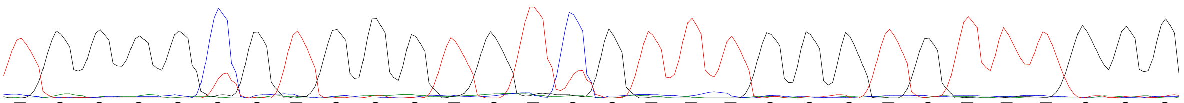

» MLiv4-bNano... (52) T G G G G C G T G G G T G T C G T T T G G G T G T T T G G G

T G G G G C G T G G G T G T C G T T T G G G T G T T T G G G

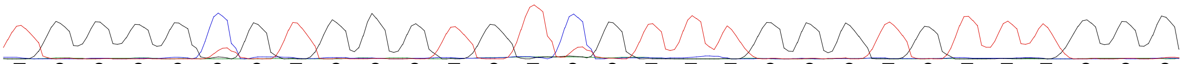

Contig 3 (52) T G G G G C G T G G G T G T C G T T T G G G T G T T T G G G

Section 2

82 90 100 111

» Reference-bN... (76) A G A A T A G G G G G T G G G T A G G G T A G G A G G T T T

» BLiv4-bNanog... (76) A G A A T A G G G G G T G G G T A G G G T A G G A G G T T T

A G A A T A G G G G G T G G G T A G G G T A G G A G G T T T

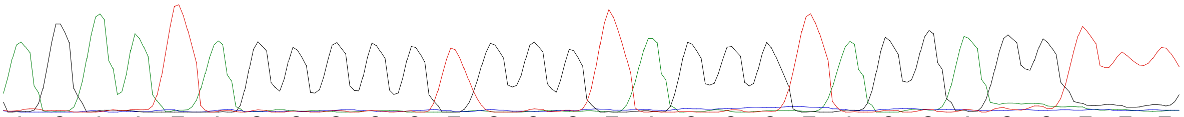

» MLiv4-bNano... (82) A G A A T A G G G G G T G G G T A G G G T A G G A G G T T T

A G A A T A G G G G G T G G G T A G G G T A G G A G G T T T

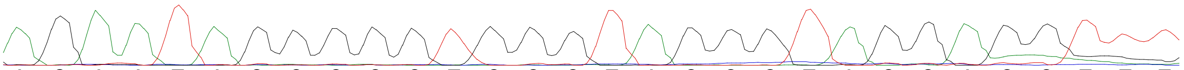

Contig 3 (82) A G A A T A G G G G G T G G G T A G G G T A G G A G G T T T

Section 3

112 120 130 141

» Reference-bN... (106) G A G G G G G G A G G A G T A G G A T T T A T T T T T T A A

» BLiv4-bNanog... (106) G A G G G G G G A G G A G T A G G A T T T A T T T T T T A A

G A G G G G G G A G G A G T A G G A T T T A T T T T T T A A

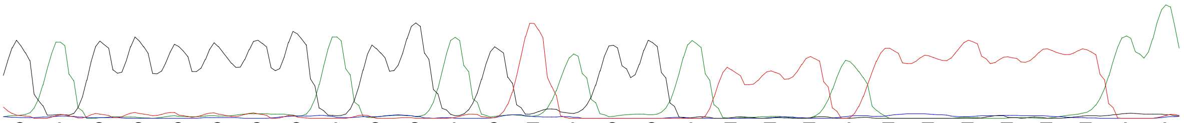

» MLiv4-bNano... (112) G A G G G G G G A G G A G T A G G A T T T A T T T T T T A A

G A G G G G G G A G G A G T A G G A T T T A T T T T T T A A

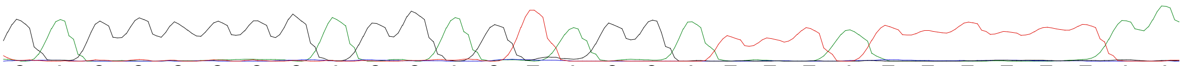

Contig 3 (112) G A G G G G G G A G G A G T A G G A T T T A T T T T T T A A

Section 4

142 150 160 171

» Reference-bN... (136) A T T T A T C G T T T
» BLiv4-bNanog... (136) A T T T A T C G T T T
A T T T A T C G T T T

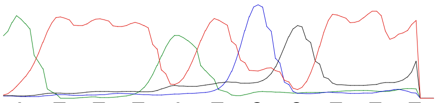

» MLiv4-bNano... (142) A T T T A T C G T T T
A T T T A T C G T T T

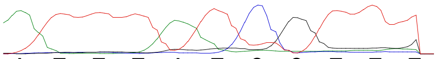

Contig 3 (142) A T T T A T C G T T T
