# Supplementary figures and images for: Epigenetic Basis of Regeneration: Analysis of Genomic DNA Methylation Profiles in the MRL/MpJ Mouse
Source: DNA Res. 2013 Aug 8;20(6):605–21. doi: 10.1093/dnares/dst034 (PMC3859327; doi:10.1093/dnares/dst034)

## Section 1

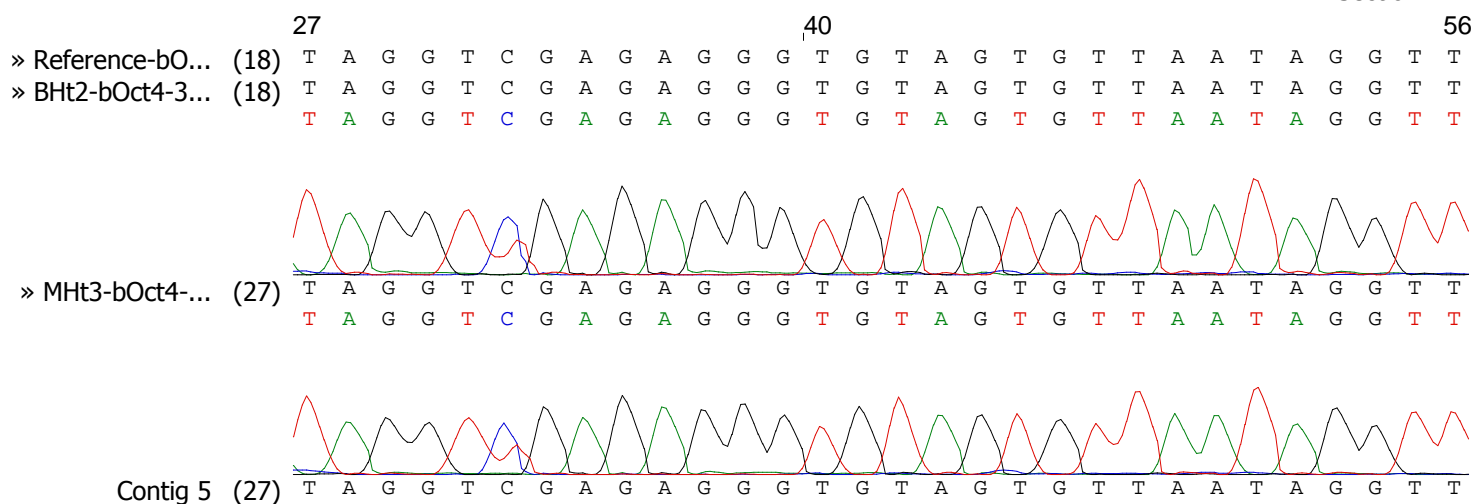

## Section 2

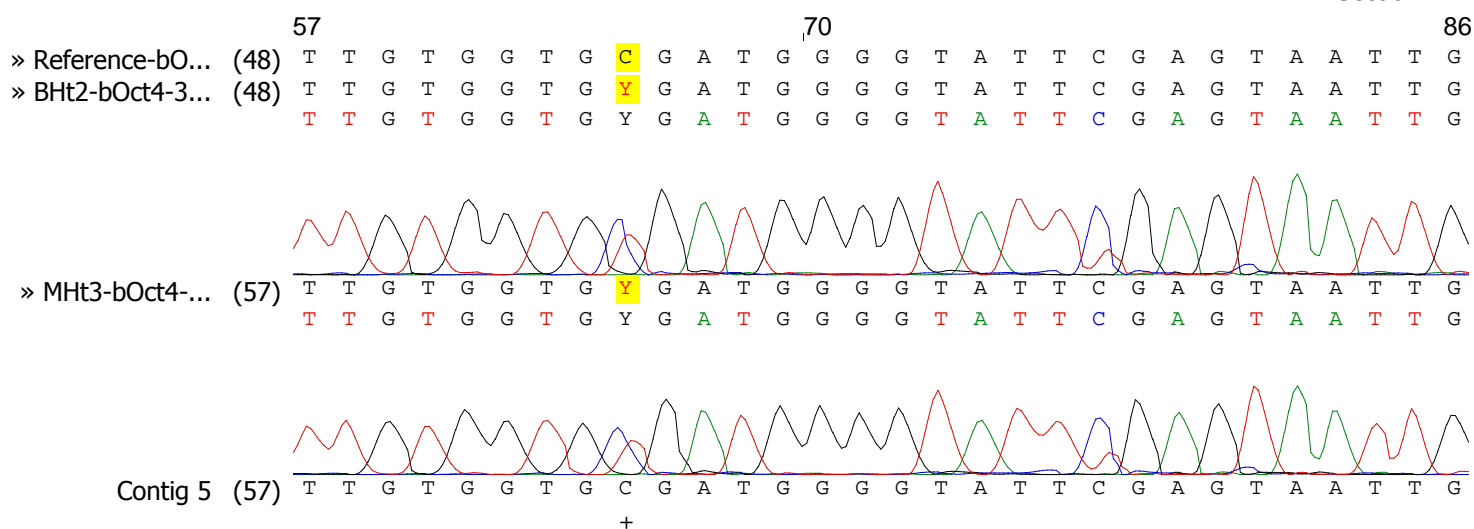

## Section 3

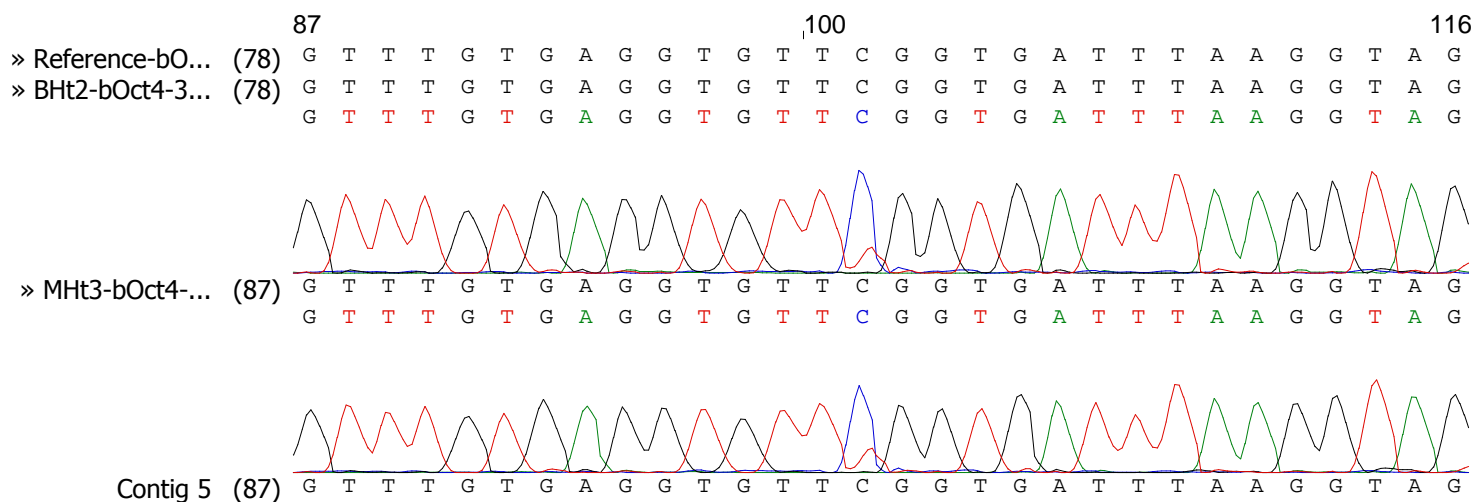

## Section 4

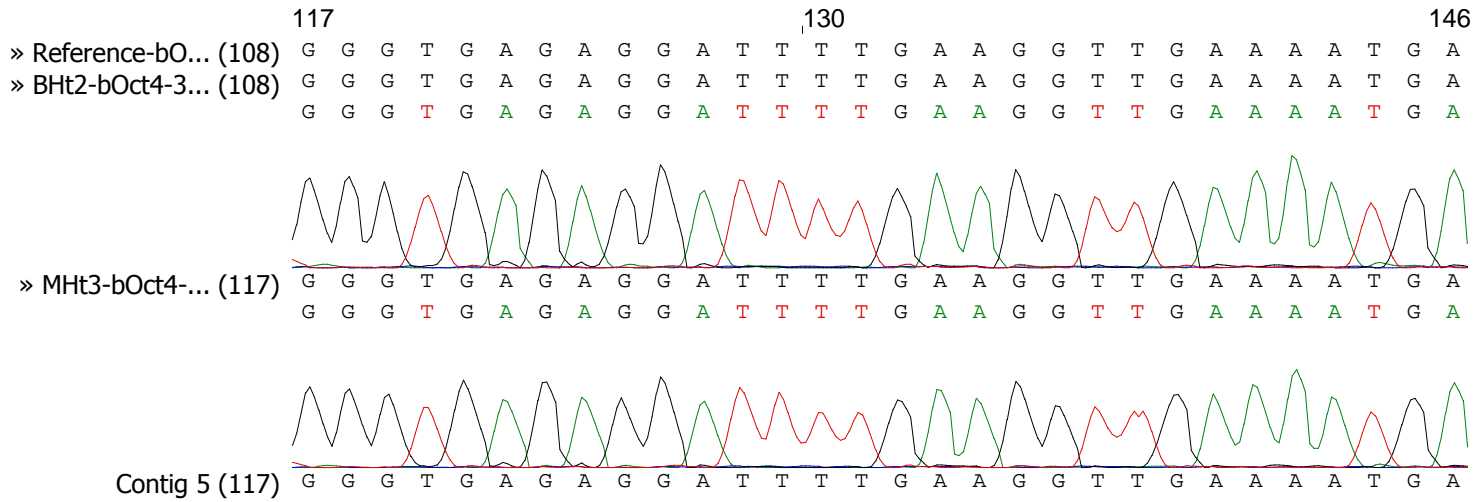

## Section 5

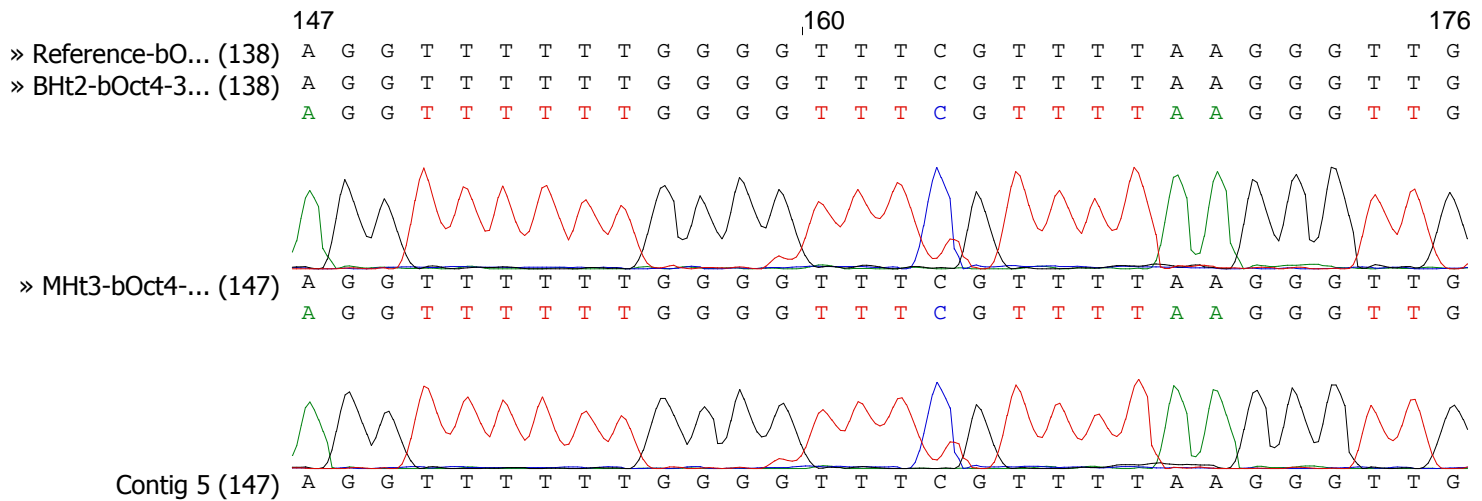

## Section 6

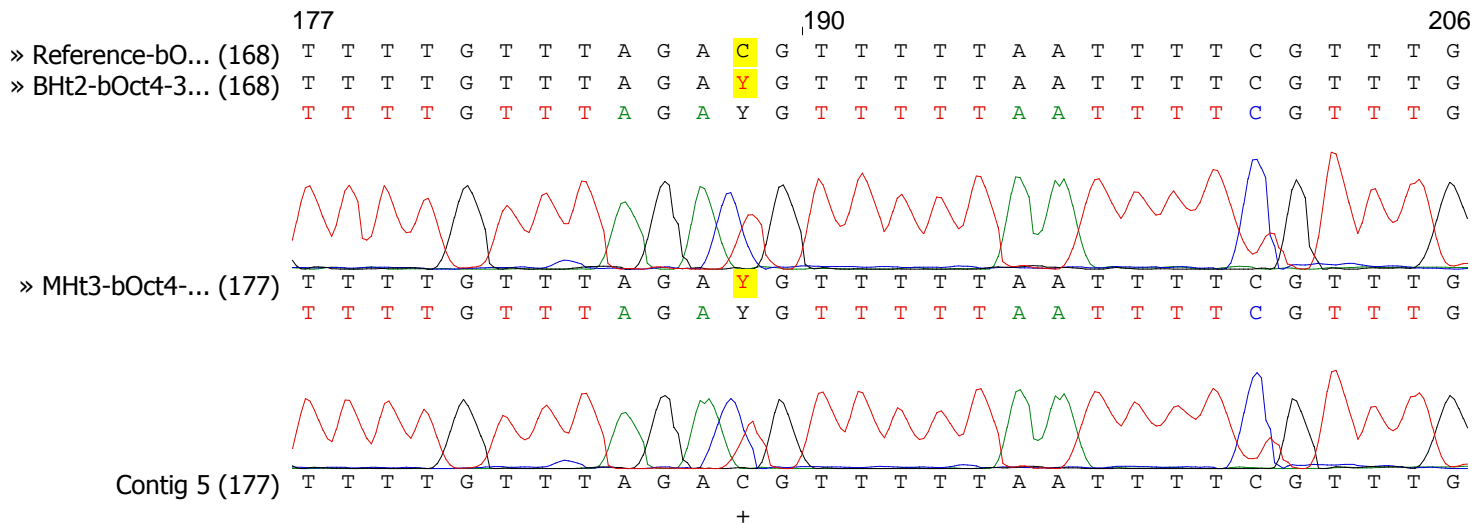

## Section 7

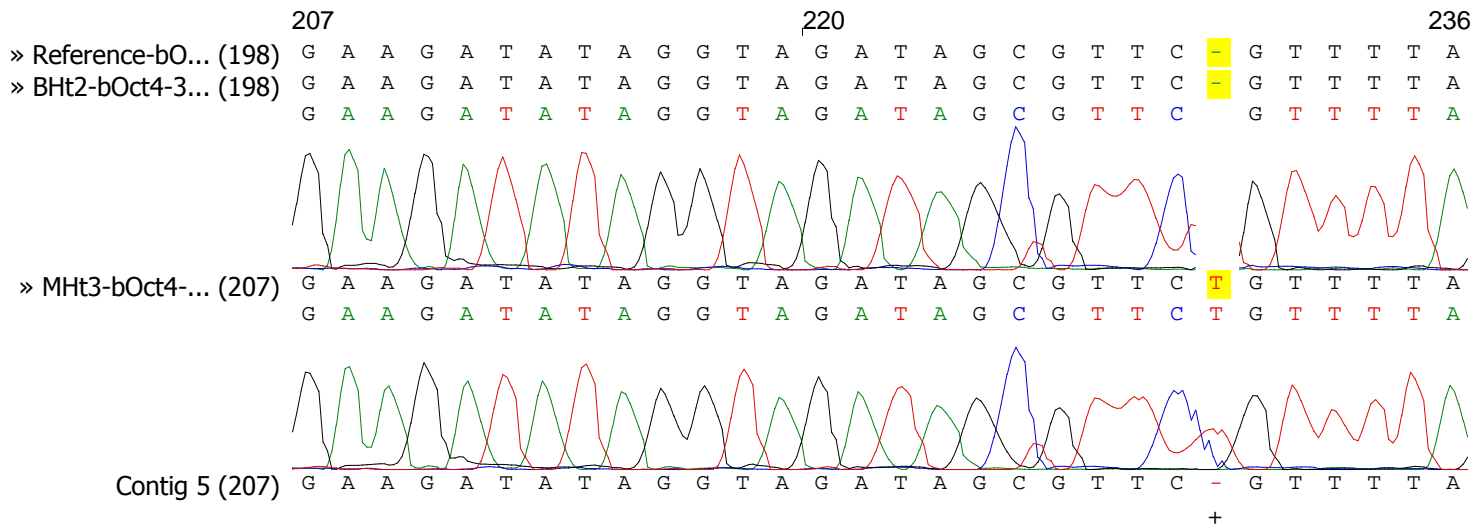

## Section 8

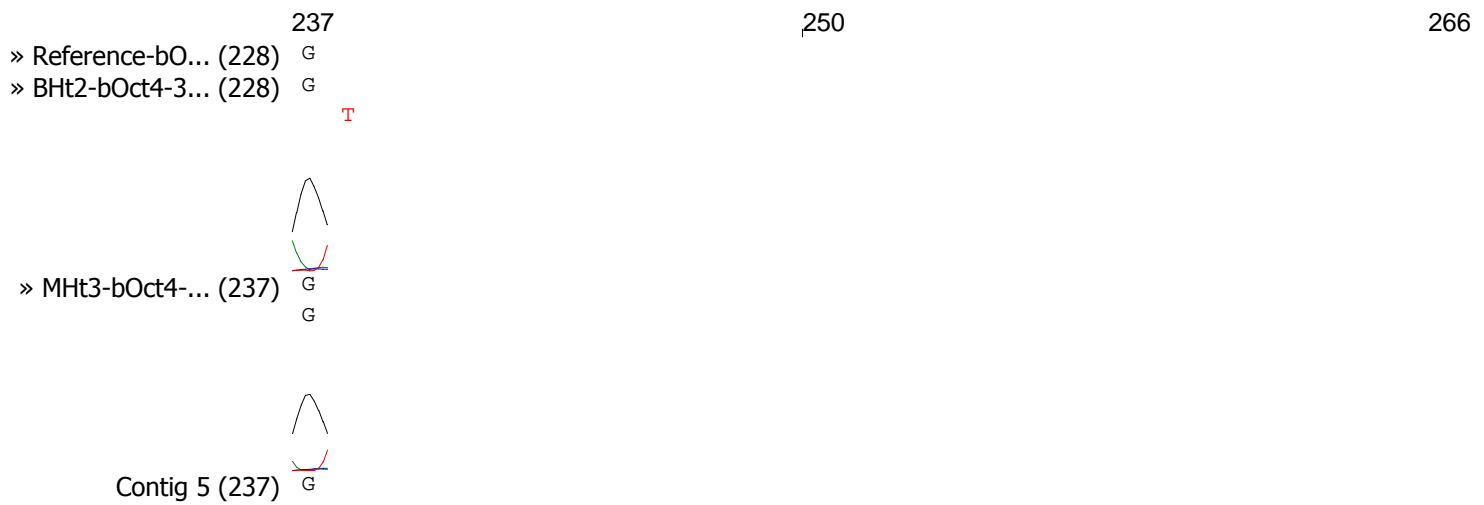

Supplement: Supplementary Data [file supp_dst034_dst034supp_data.zip › bOct4.pdf]

## Section 1

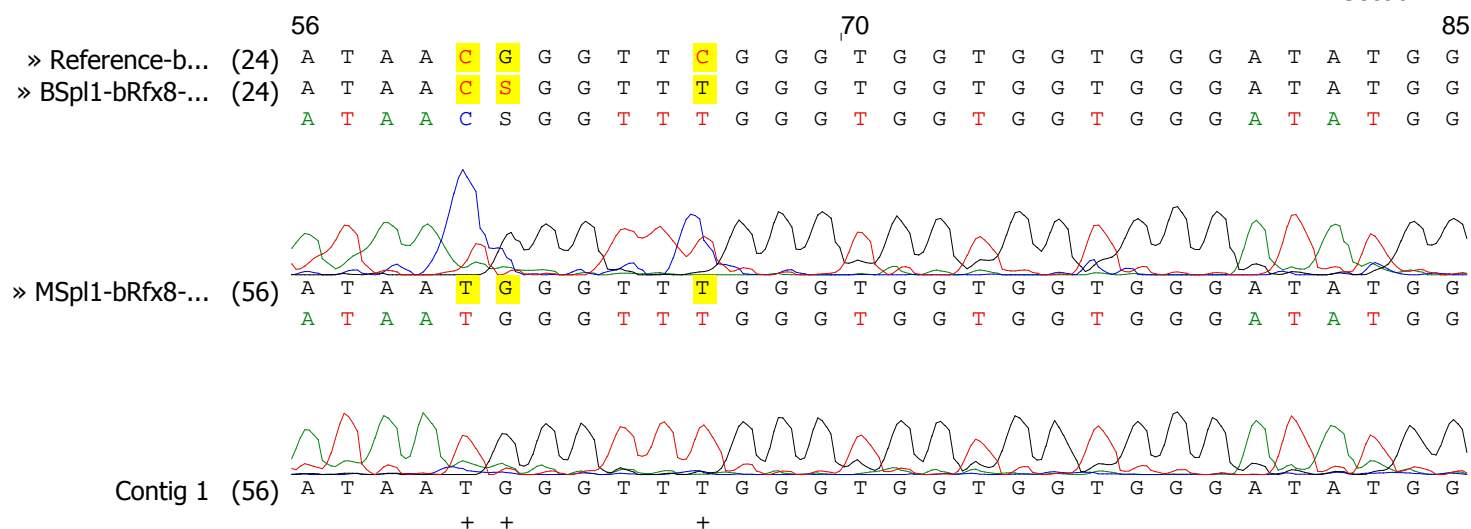

## Section 2

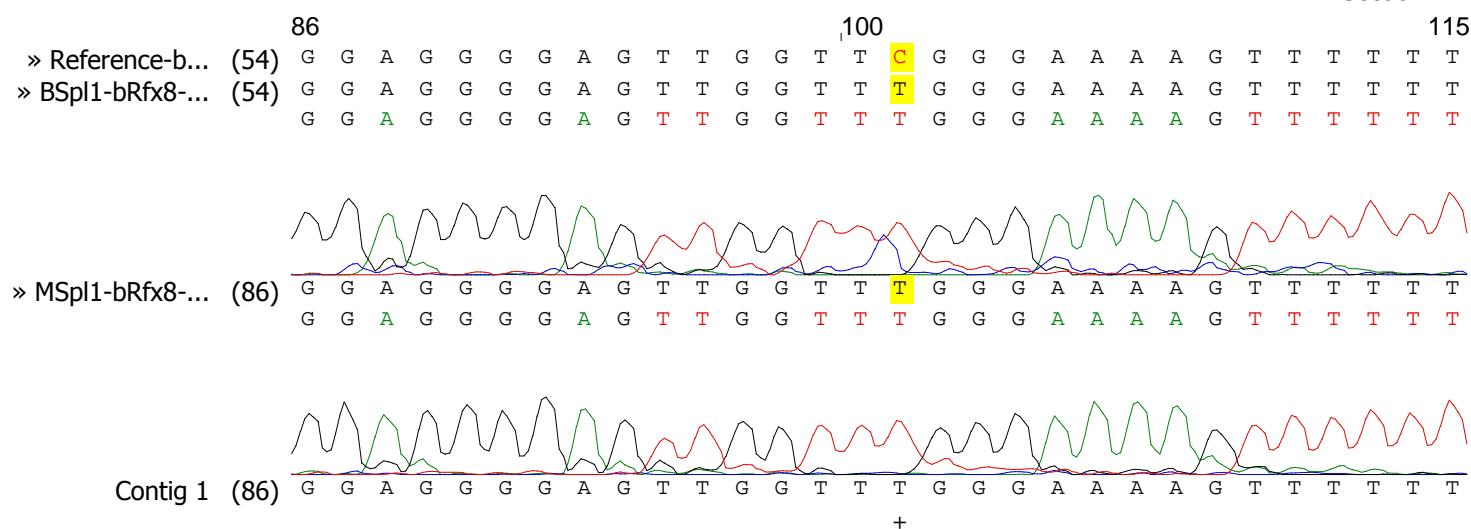

## Section 3

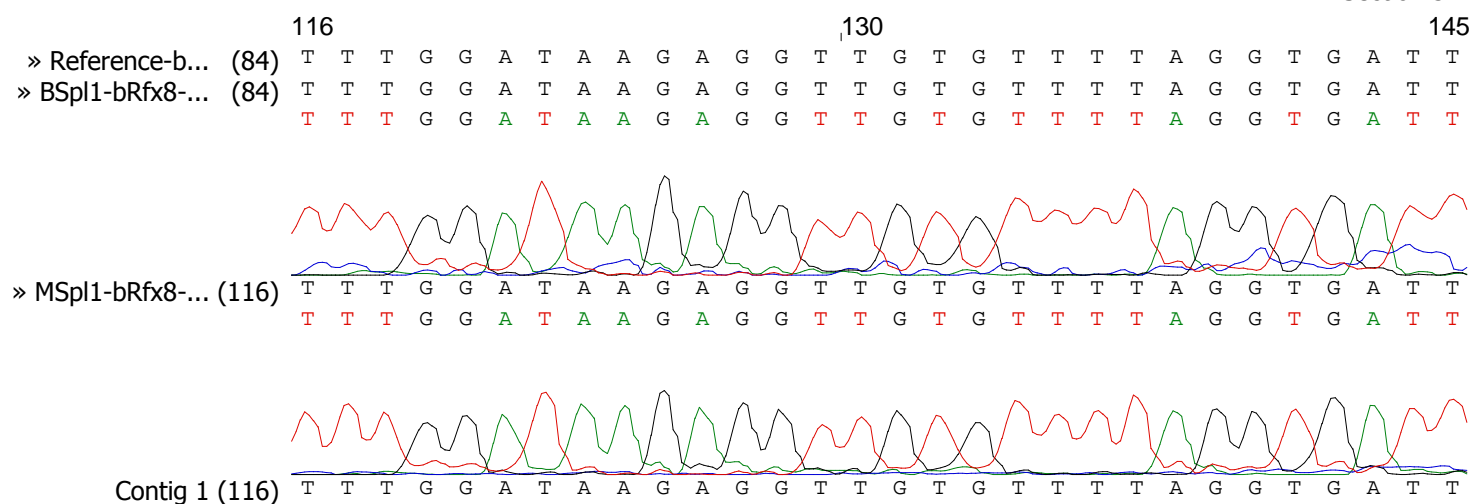

## Section 4

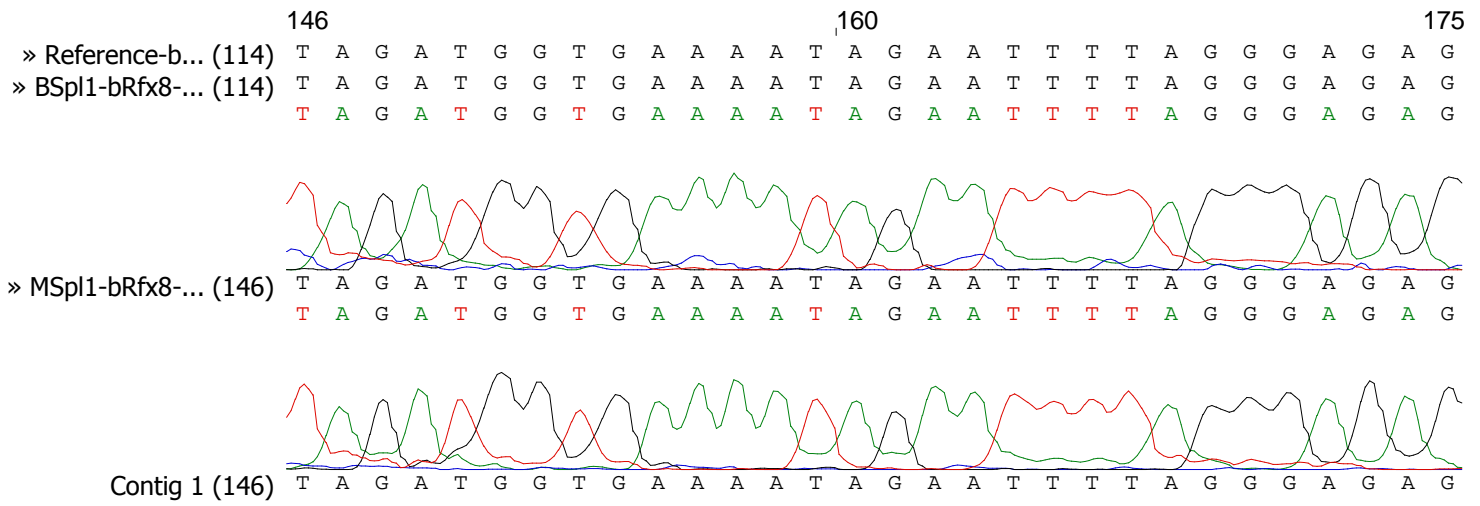

## Section 5

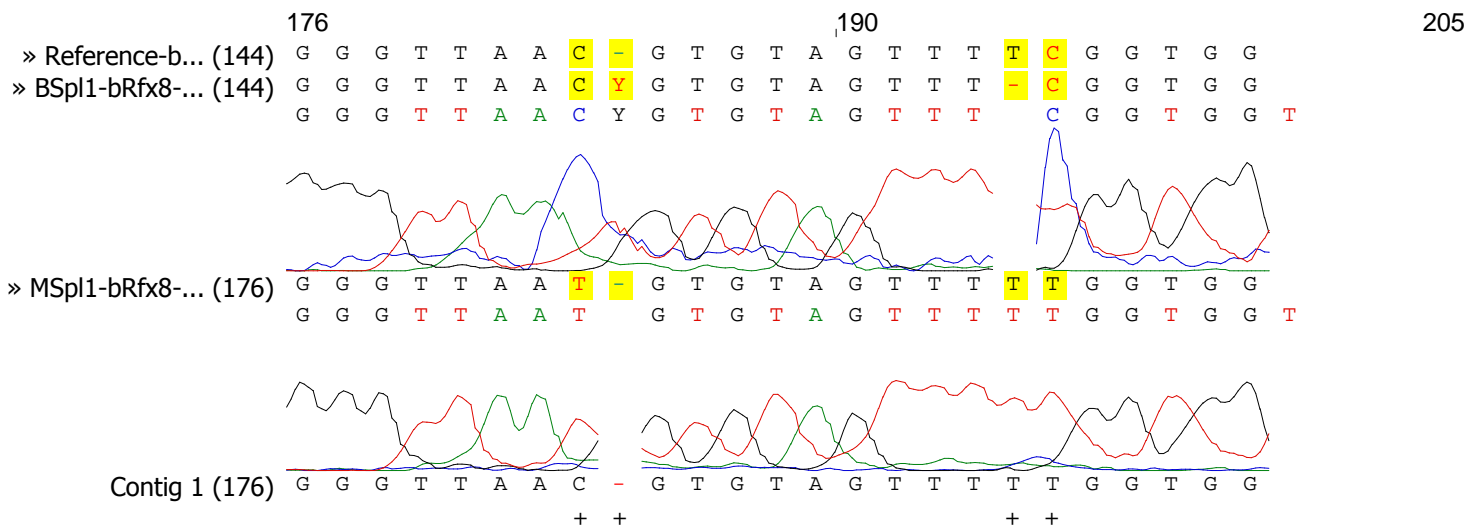

Supplement: Supplementary Data [file supp_dst034_dst034supp_data.zip › bRfx8.pdf]

## Section 1

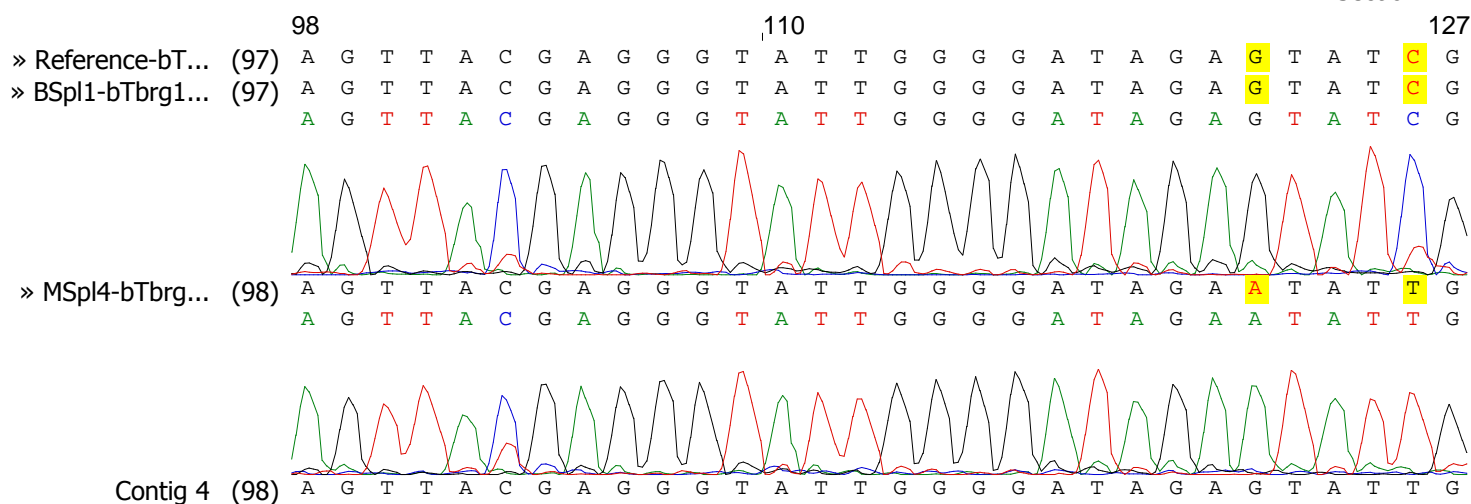

## Section 2

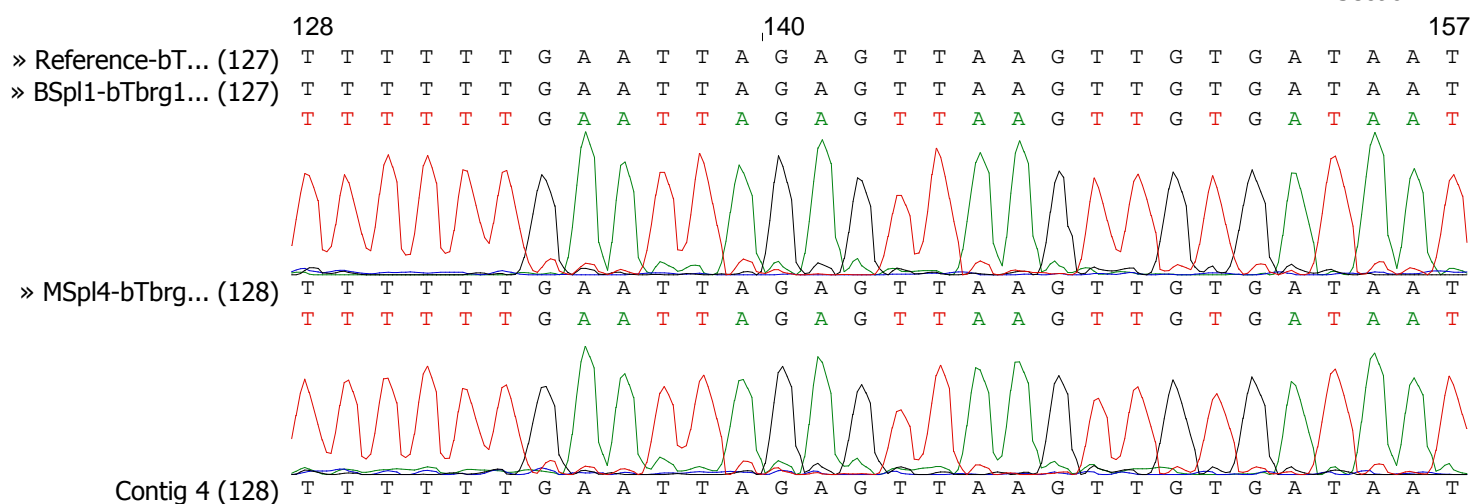

## Section 3

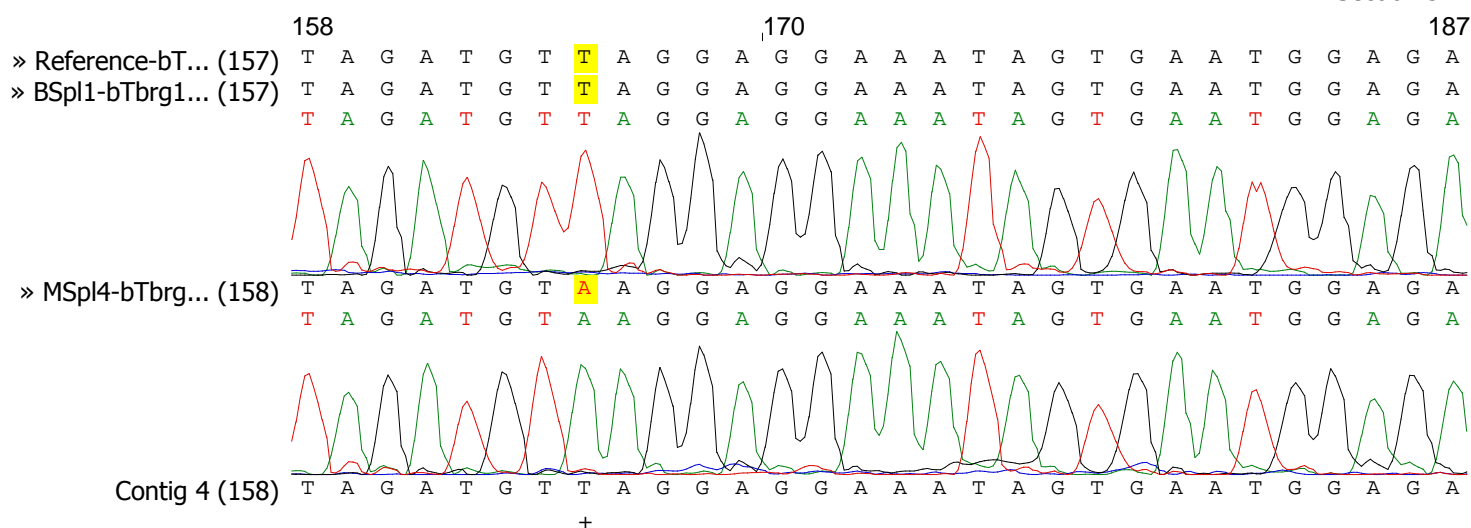

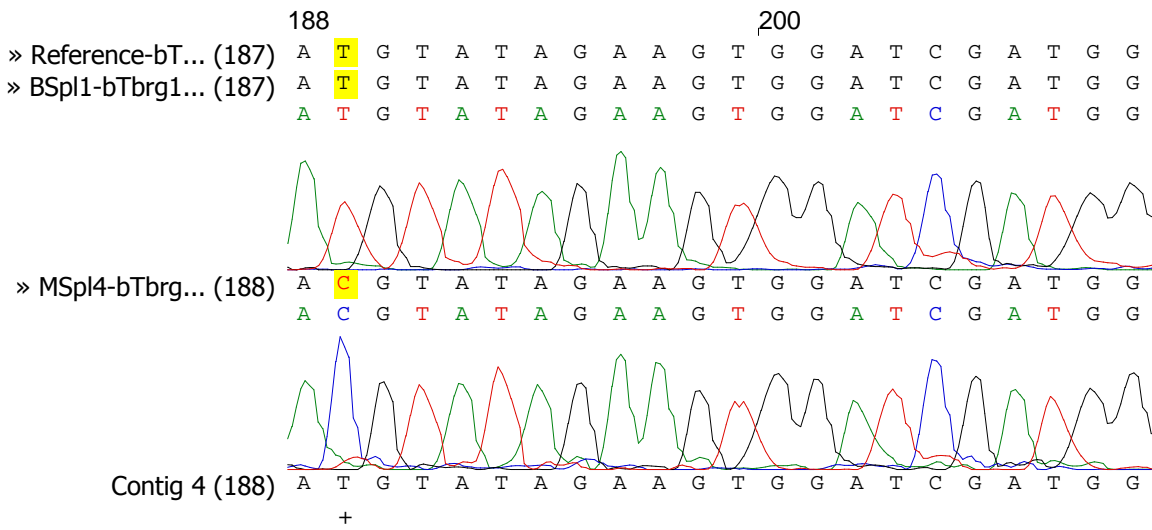

Supplement: Supplementary Data [file supp_dst034_dst034supp_data.zip › bTbrg1.pdf]
