# Supplementary material for: Epigenetic Basis of Regeneration: Analysis of Genomic DNA Methylation Profiles in the MRL/MpJ Mouse
Source: DNA Res. 2013 Aug 8;20(6):605–21. doi: 10.1093/dnares/dst034 (PMC3859327; doi:10.1093/dnares/dst034)
Supplement: Supplementary Data [file supp_dst034_dst034supp_table2.doc]

**Table S2. The list of PCR primers.**

| Gene/loci | Primer name | Sequence | Chromosomal position of the starter (MM9) | Reaction |
| --- | --- | --- | --- | --- |
| Rfx8 | gRfx8-189-F | CCAAAGGGTTCAGTATAACGG | chr1:39778197-39778219 | HpaII digestion |
| Rfx8 | gRfx8-189-R | CAGGCTTGGAGGGATCG | chr1: 39778368-39778385 | HpaII digestion |
| Rfx8 | bRfx8-254-F | ATTTTGAAAAATATTGATGATTAGTTAATA | chr1:39778158+39778188 | Bisulphite sequencing |
| Rfx8 | bRfx8-254-R | ATAAACTTCTAAAACAACTATCCCT | chr1:39778386+39778411 | Bisulphite sequencing |
| Pdgfra | gPdgfra-148-F | GTAAGCCACGGACTTGGAGA | chr5:75551644-75551664 | HpaII digestion |
| Pdgfra | gPdgfra-148-R | GTGATTCGGGGTCTGGAGTG | chr5:75551771-75551791 | HpaII digestion |
| E2f6 | gE2f6-185-F | CCGCCGCCATCCTCTCCAA | chr12:16817646-16817665 | HpaII digestion |
| E2f6 | gE2f6-185-R | GGAGGGGACCAGGGTGACCG | chr12:16817810-16817830 | HpaII digestion |
| E2f6 | tE2f6-175-F | TGCCCTTGGTCAGTGAAATAGA | chr12:16,832,461-16,832,483 | RT-qPCR |
| E2f6 | tE2f6-175-R | CACAACACCTAACTGCAAATG | chr12: 16,832,614-16,832,635 | RT-qPCR |
| CGI: chr9:63,775,406-63,775,639 | gCGI:Smad3/Smad6-108-F | GCCGCTCTGTACTTATCCTGATGA | chr9:63,775,525-63,775,549 | HpaII digestion |
| CGI: chr9:63,775,406-63,775,639 | gCGI:Smad3/Smad6-108-R | CTGCGAGAGAAGCACGGGA | chr9:63,775,613-63,775,632 | HpaII digestion |
| Akr1e1 | gAkr1e1-189-F | GTCTGGGGGAAAACCTTAATCA | chr13:4,608,187-4,608,209 | HpaII digestion |
| Akr1e1 | gAkr1e1-189-R | GGAACCATGGAAAACATCCCTA | chr13:4,608,323-4,608,375 | HpaII digestion |
| Akr1e1 | bAkr1e1-234-F | TTTTAGGATTATAGGAGAGAGAGTTT | chr13:4,608,144-4,608,170 | Bisulphite sequencing |
| Akr1e1 | bAkr1e1-234-R | AAAAAACCATAAAAAACATCCCTA | chr13:4,608,353-4,608,377 | Bisulphite sequencing |
| Akr1e1 | tAkr1e1-134-F | TTACTTATACCACAATGAGAGC | chr13:4,602,007-4,602,029 | RT-qPCR |
| Akr1e1 | tAkr1e1-134-R | TTTTCACCAATGACTTCTTGTG | chr13: 4,606,711-4,606,733 | RT-qPCR |
| Tbrg1 | bTbrg1-271-F | TTTTTTTATTATTTATATGAAGAGGGAT | chr9:37,467,581-37,467,609 | Bisulphite sequencing |
| Tbrg1 | bTbrg1-271-R | CCAAATTACCTTCTAATCTCCATACA | chr9:37,467,825-37,467,851 | Bisulphite sequencing |
| Tbrg1 | tTbrg1-339-F | CAGAATACCATCGTCGGCTCC | chr9:37,457,015-37,457,036 | RT-qPCR |
| Tbrg1 | tTbrg1-339-R | TTCGTGTGTCAGGAACTCTGGC | chr9:37,458,923-37,458,945 | RT-qPCR |
| Greb1 | tGreb1-116-F | TCCTTACGCTCCAACACCCTG | chr12:16,742,041-16,742,062 | RT-qPCR |
| Greb1 | tGreb1-116-R | TCTTCTTCCTCATTGTCTGCTCG | chr12:16,746,756-16,746,779 | RT-qPCR |
| Nanog | bNanog-209F | TGGTGGATTTTGTAGGTGGGATT | chr6:122657450-122657473 | Bisulphite sequencing |
| Nanog | bNanog-209-R | CAACCTTCCCACAAAAAAAACAAAAC | chr6:122657632-122657658 | Bisulphite sequencing |
| Pou5f1 | bOct4-315-F | CAACCTTCCCACAAAAAAAACAAAAC | chr17:35642654-35642680 | Bisulphite sequencing |
| Pou5f1 | bOct4-315-R | CAATCCCACCCTCTAACCTTA | chr17:35642947-35642968 | Bisulphite sequencing |
| Actb | tActb-500-F | TCAGAAGGACTCCTATGTGG | chr5:143666182-143666202 | RT-qPCR |
| Actb | tActb-500-R | TCTCTTTGATGTCACGCACG | chr5:143667115-143667135 | RT-qPCR |
| - | Oligo-dT_20_ | (dT)_20_ | - | Reverse Transcription |
