# Supplementary material for: Epigenetic Basis of Regeneration: Analysis of Genomic DNA Methylation Profiles in the MRL/MpJ Mouse
Source: DNA Res. 2013 Aug 8;20(6):605–21. doi: 10.1093/dnares/dst034 (PMC3859327; doi:10.1093/dnares/dst034)
Supplement: Supplementary Data [file supp_dst034_dst034supp_table3.doc]

T**able S3. The summary of microarray validation results.**

DNA methylation peaks were validated in the MRL/MpJ (MRL) and the C57BL/6J (B6) reference using bisulphite sequencing and/or digestion with a CpG methylation sensitive restriction endonuclease *Hpa*II followed by quantitative Real-Time PCR analysis (Supplemental data: Table S3 and File S1 and File S2) Gene expression levels of selected genes were examined by quantitative Real-Time PCR analysis (Table S3). The PCR primers are listed in Table S4.

| Gene/Loci | Peak location (strain) examined regon | Tissue | Microarray Resuts (Log2 peak value) | | Method (no. of samples) | Validation Results (DNA methylation: *HpaII* digested/INUPT; Gene expression: gene/*Actb*) Ratio: SEM(±SD) | | Comment |
| --- | --- | --- | --- | --- | --- | --- | --- | --- |
|  |  |  | MRL | B6 |  | MRL | B6 |  |
| *Rfx8* | chr1:39,777,740-39,778,705 (B6) | Spleen | 0 | 3.88 | *HpaII* digestion + RT-qPCR (7) | 0.043(±0.010) | 0.203(±0.044) | **p<0.05**; three *HpaII* sites |
|  |  |  |  |  | Bisulphite sequencing (1) | - | - | No C signal in MRL |
| *Pdgfra* | chr5:75,551,451-75,552,518 (B6) | Spleen | 0 | 2.67 | *HpaII* digestion + RT-qPCR (7) | 0.0275(±0.0124) | 0.0548(±0.0132) | **p<0.05**; one *HpaII* site |
| *E2f6* | chr12:16,817,374-16,817,823 (B6) | Liver | 0 | 3.26 | *HpaII* digestion (6) | 0.0058(±0.0037) | 0.0180(±0.0060) | **p<0.05**; four *HpaII* sites |
|  |  |  |  |  | RT-qPCR (3) | 0.0103(±0.0015) | 0.0055(±0.0006) | p<0.05 |
| CGI: *Smad3/Smad6* | chr9:63,775,406-63,775,639 (MRL) | Spleen | 4.16 | 0 | *HpaII* digestion + RT-qPCR (6) | 0.00840(±0.00371) | 0.00196(±0.00048) | **p<0.05**; three *HpaII* sites |
| *Akr1e1* | chr13:4,607,703-4,608,482 (MRL) | Heart | 4.58 | 0 | *HpaII* digestion + RT-qPCR (3) | 0.366(±0.041) | 0.073(±0.076) | **p<0.05**; three *HpaII* sites |
|  |  |  |  |  | Bisulphite sequencing (1) | - | - | No C signal in B6 |
|  |  |  |  |  | RT-qPCR (3) | 0.00225(±0.00165) | 0.00667(±0.00065) | **p<0.05** |
| *Tbrg1* | chr9:37,467,279-37,467,817 (MRL) | Spleen | 2.71 | 0 | Bisulphite sequencing (3) | - | - | Sequence variant( T to C substitution) The CpG is methylated in the MRL |
|  |  |  |  |  | RT-qPCR (3) | 0.258(±0.097) | 0.208(±0.075) | Non-significant |
| *Greb1* | chr12:16,810,065-16,810,611 (MRL) | Heart | 2.07 | 0 | RT-qPCR (3) | 0.000021(±0.000006) | 0.000100(±0.000041) | Non-significant |
| *Nanog* | chr6:122657450-122657658*-- | Heart Liver Spleen | - | - | Bisulphite sequencing (4 for each tissue) | - | - | No significant differences in DNA methylation |
| *Oct4* | [chr17:35642654-35642968](http://genome.ucsc.edu/cgi-bin/hgTracks?hgsid=337464153&db=mm9&position=chr17:35642654-35642968&hgPcrResult=pack)** | Heart Liver Spleen | - | - | Bisulphite sequencing (4 for each tissue) | - | - | No significant differences in DNA methylation |

Region selection based on literature:

* Hattori *et al.* Epigenetic regulation of Nanog gene in embryonic stem and trophoblast stem cells, Genes Cells. 2007 Mar;12(3):387-96.

** Hattori *et al.* Epigenetic Control of Mouse Oct-4 Gene Expression in Embryonic Stem Cells and Trophoblast Stem Cells, J Biol Chem. 2004 Apr 23;279(17):17063-9. Epub 2004 Feb 4.
