# Supplementary material for: Epigenetic Basis of Regeneration: Analysis of Genomic DNA Methylation Profiles in the MRL/MpJ Mouse
Source: DNA Res. 2013 Aug 8;20(6):605–21. doi: 10.1093/dnares/dst034 (PMC3859327; doi:10.1093/dnares/dst034)
Supplement: Supplementary Data [file supp_dst034_dst034supp_table5.doc]

| **Category** | **Terms identified by Gene Set Enrichment Analysis with p-value < 0.05** | **Number of associated genes** | | |
| --- | --- | --- | --- | --- |
|  |  | **Heart** | **Liver** | **Spleen** |
| SP_PIR_KEYWORDS | transcription regulation | 25 | **193** | **69** |
| GOTERM_MF_FAT | GO:0030528~transcription regulator activity | 20 | **140** | **56** |
| GOTERM_MF_FAT | GO:0003677~DNA binding | 26 | **199** | **70** |
| SP_PIR_KEYWORDS | dna-binding | 22 | **162** | **60** |
| SP_PIR_KEYWORDS | Transcription | 26 | **194** | **68** |
| SP_PIR_KEYWORDS | phosphoprotein | **80** | **504** | **164** |
| GOTERM_BP_FAT | GO:0045449~regulation of transcription | 30 | **225** | **79** |
| GOTERM_MF_FAT | GO:0003700~transcription factor activity | 14 | **101** | **41** |
| GOTERM_BP_FAT | GO:0006350~transcription | 26 | **192** | **66** |
| GOTERM_BP_FAT | GO:0006355~regulation of transcription, DNA-dependent | 22 | **133** | **54** |
| GOTERM_BP_FAT | GO:0051252~regulation of RNA metabolic process | 22 | **136** | **54** |
| GOTERM_BP_FAT | GO:0009890~negative regulation of biosynthetic process | 12 | **45** | **23** |
| GOTERM_BP_FAT | GO:0031327~negative regulation of cellular biosynthetic process | 12 | **45** | **23** |
| SP_PIR_KEYWORDS | alternative splicing | **61** | **323** | **110** |
| GOTERM_BP_FAT | GO:0010558~negative regulation of macromolecule biosynthetic process | 10 | **43** | **21** |
| SP_PIR_KEYWORDS | repressor | 12 | **53** | **18** |
| GOTERM_BP_FAT | GO:0010629~negative regulation of gene expression | 9 | **44** | **20** |
| GOTERM_BP_FAT | GO:0016481~negative regulation of transcription | 9 | **41** | **19** |
| GOTERM_BP_FAT | GO:0045934~negative regulation of nucleobase, nucleoside, nucleotide and nucleic acid metabolic process | 9 | **41** | **19** |
| GOTERM_BP_FAT | GO:0051172~negative regulation of nitrogen compound metabolic process | 9 | **41** | **19** |
| GOTERM_BP_FAT | GO:0010605~negative regulation of macromolecule metabolic process | 10 | **48** | **22** |
| UP_SEQ_FEATURE | splice variant | **61** | **321** | 108 |
| GOTERM_BP_FAT | **GO:0048562~embryonic organ morphogenesis** | 6 | **23** | 9 |
| UP_SEQ_FEATURE | compositionally biased region:Poly-Ser | 10 | **45** | 15 |
| UP_SEQ_FEATURE | zinc finger region:C2H2-type 3 | 7 | 29 | 10 |
| **Number of genes in analysis** | | **180** | **1063** | **336** |

**Table S5. Metabolic processes and protein families associated with the genes hypomethylated in the MRL/MpJ mouse.**

The gene set enrichment terms were identified using DAVID for the genes hypomethylated in the MRL/MpJ (Table S1) to produce the intersections for the three examined tissues. All listed enrichment terms were statistically significant with modified Fisher’s exact test p-values <0.05. The enrichment terms with p-values <0.05 after Benjamini correction are indicated by red fields.
