# Supplementary material for: Epigenetic Basis of Regeneration: Analysis of Genomic DNA Methylation Profiles in the MRL/MpJ Mouse
Source: DNA Res. 2013 Aug 8;20(6):605–21. doi: 10.1093/dnares/dst034 (PMC3859327; doi:10.1093/dnares/dst034)
Supplement: Supplementary Data [file supp_dst034_dst034supp_table6.doc]

**Table S6. The homeobox genes and the genes involved in embryonic morphogenesis which were hypomethylated in the MRL/MpJ adult mouse.**

| **embryonic organ morphogenesis** | | | **homeobox** | | |
| --- | --- | --- | --- | --- | --- |
| heart | liver | spleen | heart | liver | spleen |
| *C630004H02Rik*  *Dlx6*  ***Foxc2***  ***Insig2***  *Myo7a*  *Prox1* | ***Foxc2***  ***Insig2***  *3110039M20Rik*  *C630004H02Rik*  *Dlx2*  *Foxe1*  *Foxl2*  *Gbx2*  *Hlx*  *Hoxb2*  *Hoxb3*  *Mafb*  *Nckap1*  *Rarg*  *Rdh10*  *Satb2*  *Shh*  *Six2*  *Six4*  *Sobp*  *Tcfap2a*  *Tgfbr1*  *Tshz1* | ***Foxc2***  *Hlx*  *Hmx3*  *Hoxa7*  ***Insig2***  *Mafb*  *Rdh10*  *Satb2*  *Shh* | *Dlx6*  *Lhx6*  *Prox1* | ***Adnp***  ***Barhl1***  ***En2***  ***Esx1***  *Evx1*  ***Hlx***  *Hmx3*  *Hoxa7*  *Lass2*  *Lbx2*  *Lhx9*  *Lmx1a*  ***Mnx1***  *Onecut1*  *Onecut3*  *Pitx1*  *Pou2f1*  ***Satb1***  ***Satb2***  ***Uncx*** | ***Adnp***  ***Barhl1***  *Barhl2*  *Dlx2*  *Emx2*  ***En2***  ***Esx1***  *Gbx1*  *Gbx2*  *Gsx1*  ***Hlx***  *Hmx1*  *Hoxa10*  *Hoxa13*  *Hoxb13*  *Hoxb2*  *Hoxb3*  *Hoxc10*  *Hoxd11*  *Irx3*  *Lhx2*  *Meis1*  ***Mnx1***  *Nkx2-3*  *Nkx6-1*  *Onecut2*  *Pknox1*  *Pou3f1*  *Pou4f1*  *Pou4f2*  ***Satb1***  ***Satb2***  *Six2*  *Six4*  *Tlx3*  *Tshz1*  *Tshz3*  ***Uncx***  *Upf3b* |
